# Young children’s footwear taxonomy: An international Delphi survey of parents, health and footwear industry professionals

**DOI:** 10.1371/journal.pone.0269223

**Published:** 2022-06-09

**Authors:** Cylie M. Williams, Stewart C. Morrison, Kade Paterson, Katherine Gobbi, Sam Burton, Matthew Hill, Emma Harber, Helen Banwell

**Affiliations:** 1 School of Primary and Allied Health Care, Faculty of Medicine, Nursing and Health Science, Monash University, Frankston, Victoria, Australia; 2 Centre for Biomechanics and Rehabilitation Technologies, Staffordshire University, Staffordshire, United Kingdom; 3 School of Life Course and Population Sciences, King’s College, London, United Kingdom; 4 Centre for Health, Exercise and Sports Medicine, Department of Physiotherapy, School of Health Sciences, Faculty of Medicine Dentistry & Health Sciences, The University of Melbourne, Melbourne, Victoria, Australia; 5 Parent (Consumer Representative), Melbourne, Victoria, Australia; 6 Bobux International, Newmarket, Auckland, New Zealand; 7 Parent (Consumer Representative), Church Stretton, Shropshire, United Kingdom; 8 Allied Health and Human Performance, University of South Australia, Adelaide, South Australia, Australia; PLOS, UNITED STATES

## Abstract

**Objective:**

There is little consistency between commercial grade footwear brands for determining shoe sizing, and no universally accepted descriptors of common types or features of footwear. The primary aim of this research was to develop a footwear taxonomy about the agreed types of footwear commonly worn by children under the age of six. Secondary aims were to gain consensus of the common footwear features, when different types of footwear would be commonly worn, common terms for key footwear parts, and how movement at some of these footwear parts should be described.

**Materials and methods:**

Opinions were collected through a three-round modified Delphi international online survey from parents, health professionals, researchers, and footwear industry professionals. The first survey displayed generic pictures about different footwear types and asked participants to provide a grouping term, when the footwear would be worn (for what type of activity) and any grouping features. The second and third rounds presented consensus and gathered agreement on statements.

**Results:**

There were 121 participants who provided detailed feedback to open-ended questions. The final round resulted in consensus and agreement on the names of 14 different footwear types, when they are commonly worn and their common features. Participants also reached consensus and agreement on the terms *heel counter* to describe the back part of footwear and *fixtures* as the collective term for features allowing footwear adjustability and fastening. They also agreed on terms to quantify the flexibility at footwear sole (bend or twist) or the heel counter.

**Conclusion:**

This first taxonomy of children’s footwear represents consensus amongst different stakeholders and is an important step in promoting consistency within footwear research. One shoe does not fit all purposes, and the recommendations from this work help to inform the next steps towards ensuring greater transparency and commonality with footwear recommendations.

## Introduction

The commercial grade footwear industry has emerged as a global business, with a market reach of approximately US$360,000 million (US) in 2020, and an increasing annual growth rate of over 5% per year [1]. The footwear industry is complex, with small and large companies co-existing, often purporting design differences or mechanical properties as their ‘edge’ within a competitive market. As such, there is little consistency between commercial grade footwear brands for determinants of shoe sizing, and no universally accepted descriptors of common types or features of footwear [2]. This can be problematic when specific footwear features are desired or prescribed by health professionals as part of a therapeutic intervention, which potentially comes into conflict with any footwear benefits promoted by a footwear company.

Children’s footwear represents 18% of the commercial grade footwear sector [1] and plays an important role in protecting and supporting the growing foot [3]. This is of particular importance in the younger child, from new walkers until around 6 years of age, as they typically engage in increasingly complex bipedal activities during a time of increased tissue plasticity [4]. The purchase of children’s footwear is a common source of angst for parents and caregivers [5], with ill-fitting and poor choice of footwear often cited as the basis of foot-related issues as adults [6]. This angst can be heightened when children present with disability or developmental concerns, where specific footwear features may assist in achieving, improving or maintaining ambulation [7–10]. The lack of consistency in descriptors of footwear types and features can stymie both health professionals and parents as it is typically dependant on the individual retail centre to interpret prescribed or recommended inclusions. Additionally, this lack of established descriptors limits the ability to confidently compare research outcomes when investigating the impact of footwear given type and features cannot be robustly described [2, 7].

The primary aim of this research was to develop a footwear taxonomy through international consensus about the types of footwear commonly worn by children under the age of six. Secondary aims were to gain consensus of the common footwear features, when different types of footwear would be commonly worn, common terms for key footwear parts, and how movement at some of these footwear parts should be described.

## Materials and methods

### Design

The study was an international three-round modified Delphi online survey. This design consisted of an initial round where participants’ provided their opinion to gather consensus [11]. Any responses not reaching consensus were then returned to participants for consideration, and rating agreement in subsequent rounds. This research was approved the Monash University Human Research Ethics Committee (25698). All participants provided written informed consent through their response to the online survey.

### Participants

Participants were recruited through institutional and personal social media accounts of the authors. Participants were eligible to be part of the Delphi survey if they self-identified in any of the following categories:

1. A parent of a child/children under the age of six years and had purchased shoes for their child in a shoe store with fitting support.
2. A health professional who had made regular footwear recommendations for children under the age of six years, in the past six months.
3. A researcher who had researched young children’s footwear in the past 10 years
4. A professional who had sold footwear in the past six months for children under the age of six years.
5. A professional who had worked in footwear design for children under the age of six years in the last six months.

Advertisements to encourage participation were customized to health professionals, researchers, parents of children under the age of six, and people working in the footwear industry directly relating to footwear for young children. These were advertised on social media at weekly intervals during Round 1.

There were no geographical boundaries to recruitment. Participants checked an online consent box for ongoing communication as part of the research, and to signify their commitment to responses to all rounds. Intra-panel communication was anonymous, and participants were asked to keep their responses in each round confidential. No enticements or compensation were provided.

### Procedure

A purpose-built survey was developed by the authorship team due to the novelty of the questions of interest. Face validity was tested during development by collecting multiple photos of footwear types from those currently in online advertising in Australia, the United Kingdom and the United States. All authors then reviewed pictures of the types of footwear and agreed on grouping styles, that all grouping styles were represented, and the question phrasing for the target participants. The authorship group consisted of five clinician researchers, two parents with no research experience and one industry representative. All authors participated in all rounds of survey designs. Round 1 survey was then piloted with one parent and two health professionals and wording clarified based on their feedback.

All data were collected using the online survey platform Qualtrics® software (Qualtrics, Provo, UT, USA). Data were linked at each round through participant-provided email. Qualtrics® routinely collects Internet Protocol (IP) addresses as part of the de-identified metadata in the survey response and participants were provided with this information as part of their informed consent. The IPs were only viewed and used as a last resort (1 occasion) to match data where emails responses in subsequent rounds did not match those in the Round 1. All rounds were open for four calendar weeks and participants were reminded weekly.

Feedback to participants at each round was provided within the online survey and participants were invited to provide feedback on terminology or grammar. Final results were provided to all participants if they completed all rounds.

#### Round 1

Participants self-selected the group they identified with and were able to select more than one if it was applicable. Participants were asked to provide their gender and residing country. Based on the group selection, additional information was collected using survey software logic. This meant that only the questions relevant to the selected group were displayed. For example, if they identified as a parent, they were asked how many children they had, and the age of their youngest child. Health professionals were asked to provide their profession, how many children treated in a typical month who were aged under six years, and how long they had been working in the role. Researchers, footwear designers and those working in footwear retail were also asked how many years they had been working in their role.

**Fig 1:**
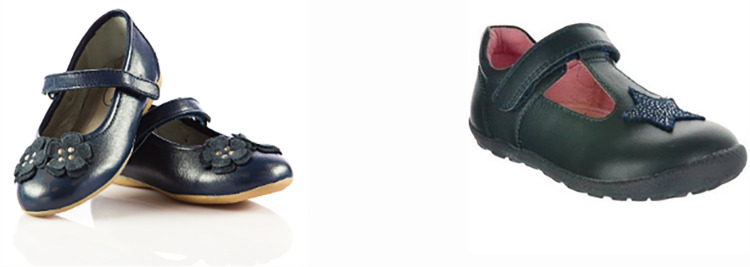
Footwear style example.

Participants were then progressed through the first round of the online survey. The survey presented participants with three pictures of similar footwear that had similar features (S1 File). These footwear pictures and their groupings were co-designed by all authors based on their expertise as consumers, health professionals or footwear designers. No brands were shown, and all footwear pictures were of shoe styles available in the countries of the authors. An example of the figures is displayed in Fig 1.

For each footwear group picture, participants were asked the following questions (and prompt for the question was placed in *italics*).

1. When you look at the pictures, what would you call this group of footwear?
  (This may be a simple response and we’d urge you to consider the first grouping word that comes to mind.)
2. When do children usually wear this type of footwear?
  (This may be related to a particular time of year, a season or seasonal activity or when a child does a particular activity where they would commonly wear this type of footwear for.)
3. What are the common features of this group of footwear?
  (We’d encourage you to be as detailed as possible, and list as many features are you can think of. Features are like how high the shoes are, what the top or bottom of the footwear looks like, as well as the front and back of the footwear.)
4. What are some of the other names you have heard these features called, with similar features to these?
  (This may be what the store calls them, what your parents, friends or interstate or international colleagues call them.)

Questions within the survey were specifically designed to not prompt any terms or infer responses for future questions.

**Fig 2:**
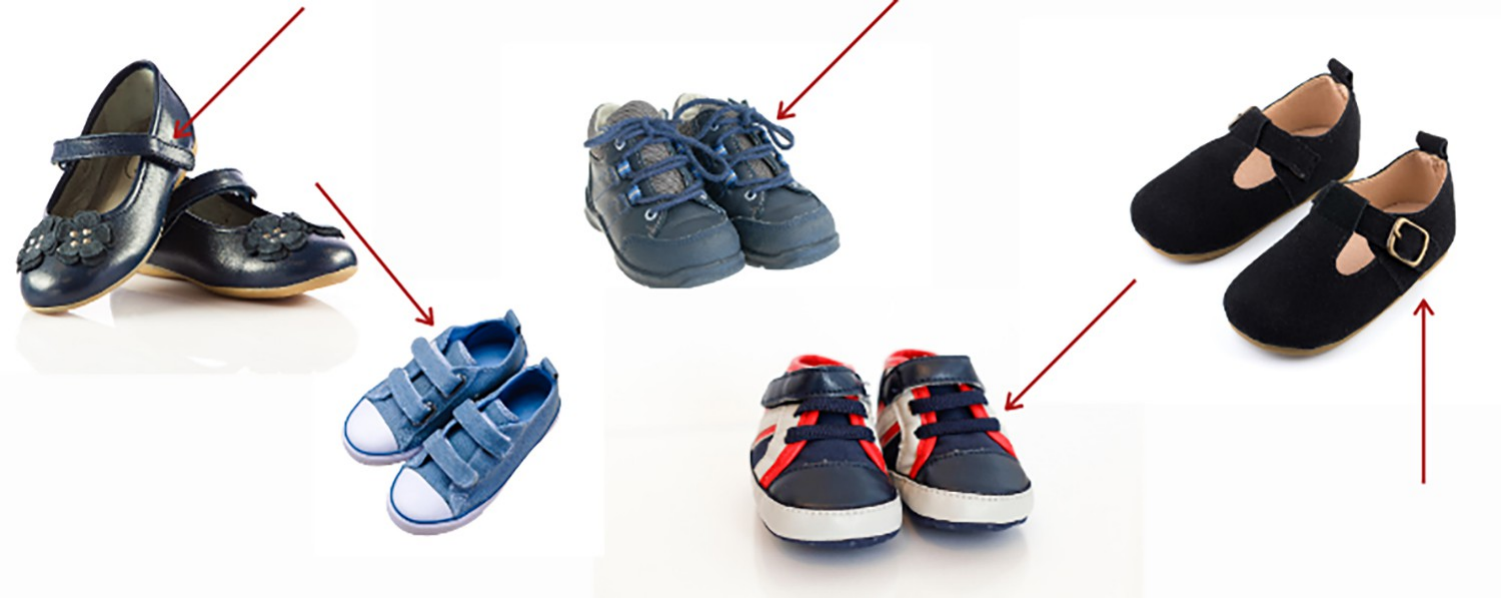
Footwear features example.

Participants were then invited to describe any other footwear types young children commonly wear that were not displayed in the pictures. Participants were shown three pictures of footwear with different responses to torsion applied to the sole of the footwear, three pictures of different responses to pressure applied at the back of the heel of the footwear and a picture of different footwear with adjustable features. Participants were asked to describe a group term for these features shown in the pictures. An example of these is shown in Fig 2.

To develop Round 2, participant responses were initially grouped into a) Health professionals and researchers, b) Parents and c) Footwear industry professionals, based on the numbers of responses. Where participants selected more than one category, they were allocated to the category on the hierarchal order based on training and exposure to footwear and where health professionals and researchers were set at the highest category. For example, if a participant responded that they were a health professional, parent and sold footwear, they were placed in the health professional grouping.

Inductive quantitative content analysis of the open questions was undertaken. This method of analysis allowed for statements and comments to be individually considered, the content of these statements based on what is commonly understood about footwear and a statement made with common themes [12]. This approach meant that the first participant’s comment was considered, and one or more statements developed from this. The next comment was then considered and counted towards that statement or a new statement generated. As anticipated, the length of statements varied, however, even where the statement was one word, it was counted to a statement or a new statement generated. This grouping took an iterative approach, whereby if a new statement emerged, earlier comments were recoded.

The data were initially analysed by a single researcher (CW). To reduce individual bias, the participant comments and statements were independently reviewed by an additional author, with all other authors reviewing at least 5 comments each. Each author provided secondary review based on their knowledge and own personal grouping (Health professionals–HB, SM, KP, MH, parents–KG, EH or footwear industry—SB). Disagreements were resolved by discussion. Reflexivity was acknowledged as a concept that introduces personal bias into research [13]. Authors analysing this data acknowledged their different individual experiences with children’s footwear, purchasing, knowledge, and how these different experiences may have influenced the analysis.

#### Round 2

Statements presented to participants in Round 2 (S2 File) were considered to have reached consensus within Round one when 70% or more of the participants in each group indicated the same statement content by agreement of two authors. This percentage was consistent with existing literature [14, 15]. Participant groupings were used to ensure equal consideration of the views of all participants for Round 1 only. This subgrouping was used to ensure one grouping did not unduly influence the results based on participant numbers.

Only statements arising from 50–69% of participants in total or within any subgroup were presented to participants in Round 2 for agreement rating. Participants were made aware when there was less than 50% of the total number of responses, but where there was a group that had a 50% or greater response. They were not informed which group reached 50% or greater so as not to influence any bias or value judgement placed on the statement. Statements where less than 50% of participants in any group responded similarly were discarded and did not appear in Round 2.

Participants were then asked to indicate their agreement with each statement on a four point Likert scale where 1 was Strongly Disagree, 2 was Disagree, 3 was Agree and 4 was Strongly Agree. They were also asked to provide suggestions to grammar or statement wording if they did not agree with the statement.

#### Round 3

Similar to the process in Round 2, statements where 70% or more participants agreed or strongly agreed were included (S3 File). It was planned that statements where less than 70% of participants agreed were discarded from the final result, consistent with the Delphi survey process.

### Analysis

Descriptive statistics and analysis of responses of each round were undertaken in Microsoft Excel 2018 (Microsoft Corp, Redmond Washington). The authors made *a priori* decision that the Delphi would conclude if the total or sub-group participant response rate dropped below 70%, or if round three was required and completed, irrespective of agreement. Participants who did not complete the entire questions in Round 1 were excluded and not invited to complete Rounds 2 and 3.

## Results

### Participants

**Fig 3:**
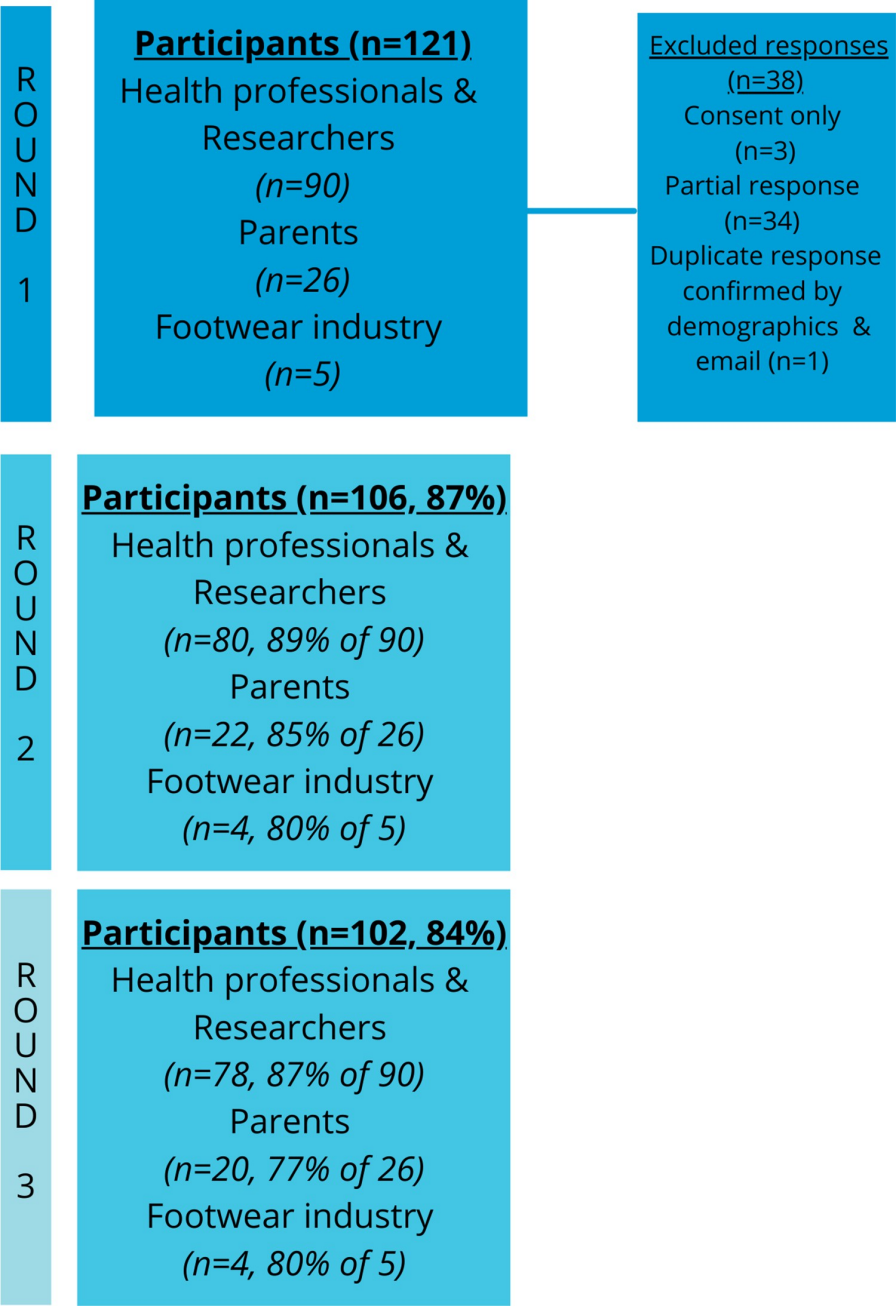
Participant flow through rounds.

There were 159 participants who consented to complete the first round of the Delphi survey. Of these, there were 121 completed responses. Demographics of included participants and their subgroupings are provided in Table 1. Table 2 provides further details about the health professionals who participated, including a breakdown of the professions, average number of young children treated per month and years of experience. The number of participants in each round is shown in Fig 3. There were 55 (45% of 121) participants who had children <6 years of age, and nine participants who worked in the footwear industry, four of these were also health professionals. The median (IQR) number of children was 2 (1, 2) and the median child age was 3 (1,4) years.

**Table 1:** Demographics of participants and allocations to subgroups.

	Total participants (N = 121)
Country, n(%)	
*Australia*	65 (54%)
*United Kingdom*	30 (25%)
*USA*	11 (9%)
*Other* *	15 (12%)
Female, n(%)	98 (91%)
Health professionals, n (%)	90 (74%)
Researchers, n (%)	1 (1%)
Working in the footwear industry	9 (7%)
Parents of children <6 years of age	55 (45%)
**Participant Grouping 1** *(Health professionals and researchers who may also sell*, *design footwear or also be parents)* n (%)	90 (74%)
**Participant Grouping 2** *(Parents only)* n (%)	26 (21%)
**Participant Grouping 3** *(People who sell and design footwear only)*, n (%)	5 (5%)

*Canada, Malta, Singapore, Denmark, New Zealand

**Table 2:** Health professional participant demographics (n = 90).

	Total health professionals (n = 90)
Podiatrist	42 (47%)
Physiotherapist or Physical Therapist	40 (44%
Orthotist or Pedorthist	8 (9%)
Number of children treated in a typical month	
*<10*	29 (33%)
*10–19*	24 (27%)
*>20*	37 (40%)
Years of experience	
<5 years	10 (11%)
5–9 years	21 (23%)
10 or more years	59 (66%)

### Consensus

Round 1 took participants approximately 60 minutes to complete. Participants generated 147 statements in response to open ended questions. There were 16 consensus statements about the names for footwear styles, when they are worn, their common footwear features. Tables 3 and 4 provide all statements generated by participants and the frequency (%) of participants who provided the same response. Statements highlighted in green were those that meet consensus (≥70% of all participants providing the same response). There were 71 statements where less than 50% of participants described content, these were discarded at Round 1, and highlighted in red in Tables 3 and 4. Statements highlighted in orange in Tables 3 and 4 were developed from similar statements from 50–69% of participants in total, or in each participant group and progressed to the next round.

**Table 3:** Generated statements about young children’s footwear taxonomy and round in which the statements were accepted.

Domain	Statement	Round 1 n (%) of 121 participant responses* n (%)^*a-90*, *b-26 or c-5*^	Round 2 (n) % of 105 participant responses	Round 3 n (%) of 102 participant responses
Style	**Boot**	114 (94)		
Worn	Boots are often worn when it is cold, wet, snowing, or in winter.	115 (95)		
Worn	Boots are often worn when going outdoors	65 (54)	96 (91)	
Worn	Boots are often worn during physical activity such as walking, hiking or climbing	48 (40) *3 (60)*^*c*^	96 (91)	
Worn	Boots are prescribed by health professional for foot support or a foot problem	30 (25)		
Feature	Boots cover the ankle	106 (88)		
Feature	Boot sole has a tread pattern with a variable heel height and/or width	37 (31)		
Feature	The boot sole is commonly made of a material that resists bending	69 (57)	84 (79)	
Feature	The boot upper material covers the toes and foot	53 (44) *45 (50)*^*a*^	100 (94)	
Feature	The boot upper material is commonly leather or a material that can be waterproofed	53 (44) *4 (80)*^*c*^	91 (86)	
Feature	Boots commonly have fastenings or elastic to improve their fit	53 (44) *45 (50)*^*a*^	86 (81)	
Feature	Boots are structured around the heel	38 (31)		
Style	**Sneaker**	62 (51)	75 (71)	
Style	Plimsol	19 (16)		
Style	Joggers	5 (4)		
Style	Runners	31 (26)		
Worn	Sneakers are commonly worn when being physically active, or for casual occasions. These activities or occasions may include play, or event-based occasions (e.g. family gatherings).	85 (70)		
Worn	Recommended by a health professional for its benefit or a foot problem	2 (2)		
Worn	Sneakers are commonly worn when the weather is dry or warm	62 (51)	87 (82)	
Worn	Sneakers are commonly worn outdoors	75 (62)	93 (88)	
Worn	Worn at a particular age or stage	28 (23)		
Feature	Sneakers commonly have a soft or very flexible sole	87 (72)		
Feature	Sneakers commonly have an upper material fully covers the top of the foot	86 (71)		
Feature	Sneakers commonly have a heel counter that has some structure and stiffness	76 (63)	75 (71)	
Feature	Sneakers commonly have fasteners such as Velcro or laces to adjust the fit to the foot	69 (57)	99 (93)	
Feature	Upper is commonly canvas or leather	57 (47)		
Feature	Light weight	15 (12)		
Feature	Footwear finished under the ankle	41 (34)		
Feature	Limited or no arch support	2 (2)		
Feature	Light weight	15 (12)		
Style	**Sneaker**	85 (70)		
Style	**Runner**	78 (64)	81 (76)	
Style	**Sport/athletic footwear**	42 (35) *3 (60)*^*c*^	98 (92)	
Style	Jogger	32 (26)^β^		
Style	Trainers	49 (40)		
Style	Sand shoes	8 (7)		
Style	Pumps/takkies or kicks	3 (2)		
Style	Brand or sport specific shoe name	20 (17)		
Worn	This style of footwear is commonly worn when being very active, such as running or playing sport	116 (96)		
Worn	This style footwear is commonly worn in all seasons	43 (36) *3 (60)*^*c*^	99 (93)	
Worn	This style of footwear is commonly worn outdoors or during organised care (e.g. nursery school or kindergarten)	67 (55)	100 (94)	
Worn	Worn on recommendation by a health professional for its benefit or features	5 (4)		
Worn	This style of footwear can be worn everyday	40 (33) *3 (60)*^*c*^	98 (92)	
Worn	Worn at a particular age or developmental stage	3 (2)		
Feature	This style of footwear has semi-flexible sole made of cushioned material	82 (68)	88 (83)	
Feature	The bottom or sole of this type of footwear has a gripping tread, and is higher underneath the bottom of the heel area than underneath the front area	57 (47) 46 (51)	90 (85)	
Feature	The upper material of this style of footwear covers the top of the foot	64 (53)	101 (95)	
Feature	This style of footwear has fasteners to adjust fit	79 (65)	99 (93)	
Feature	Footwear finishes under the ankle	35 (30)		
Feature	The footwear is light weight	10 (8)		
Feature	The material of the upper has features to improve breathability/airflow	44 (36)		
Feature	This style of footwear commonly has a structured and semi-flexible heel counter	77 (64)	99 (93)	
Feature	The footwear has an insole that is moulded or has an arch contour	23 (19)		
Style	**Sandal**	100 (83)		
Style	Summer shoes	13 (11)		
Style	Miscellaneous terms such as Slip ons, jandals, jellies, beach shoes, open toe shoes, thongs, slides	25 (21)		
Worn	Sandals are commonly worn during summer, or in warm weather	98 (81)		
Worn	Sandals are commonly worn outside to places like the beach, or for casual outings	74 (61)	97 (92)	
Worn	Worn for particular play activities such as wet play or outdoor play	24 (20)		
Worn	Worn at particular ages or for every day	3 (2)		
Feature	Sandals commonly have upper material that has gaps or holes, and the material may or may not totally cover the toes	97 (80)		
Feature	Sandals commonly have a semi flexible flat sole	56 (46) *3 (60)*^*c*^	88 (83)	
Feature	Sandals can have either a strap at the heel or an enclosed back	69 (57)	100 (94)	
Feature	The upper material of sandals is commonly either leather or synthetic material	23 (19) *3 (60)*^*c*^	101 (95)	
Feature	Sandals commonly have a strap around the front of the ankle that can be adjusted for fit	78 (64)	95 (90)	
Feature	Footwear finishes under the ankle	18 (15)		
Feature	The footwear is light weight	7 (6)		
Style	**Pre-walkers**	52 (43) *4 (80)*^*c*^	94 (89)	
Style	Booties	20 (22)		
Style	**Soft-soled footwear**	19 (16) *5 (100)*^*c*^	96 (91)	
Style	Moccasins	16 (13)		
Style	Baby shoes	50 (41)		
Style	Slippers	28 (23)		
Worn	Pre-walkers or soft-soled footwear can be worn by babies or children not yet confidently walking	73 (60)	98 (92)	
Worn	Footwear can be worn as a fashion accessory or item	12 (10)		
Worn	Pre-walkers or soft-soled footwear can be worn indoors or during organised care (i.e. Nursery or daycare)	26 (21) *3 (60)*^*c*^	92 (87)	
Worn	Pre-walkers or soft-soled footwear can be worn while learning a new skill such as crawling or walking	48 (40) *3 (60)*^*c*^	83 (78)	
Worn	Pre-walkers or soft-soled footwear can protect feet from the environment or the cold	37 (31) *3 (60)*^*c*^	98 (92)	
Feature	Pre-walker or soft-soled footwear has a soft and fully flexible sole	111 (92)		
Feature	The upper material and heel area (heel counter) of pre-walkers or soft-soled footwear are fully flexible	101 (83)		
Feature	The upper of pre-walkers or soft-soled footwear is either made of leather, fabric or a synthetic material that is soft.	3 (26) *3 (60)*^*c*^	101 (95)	
Feature	Footwear finishes under the ankle	8 (7)		
Feature	Footwear has some form of fixation to keep the shoe on the foot	18 (15)		
Feature	Footwear commonly slips onto the foot	18 (15)		
Style	**Mary-Jane**	80 (66)	86 (91)	
Style	**T-Bar**	25 (21) *3 (60)*^*c*^	68 (64)	71 (70)
Style	Dress shoe	30 (25)		
Style	Court. Formal, dolly or party shoe	13 (11)		
Style	Ballet flats or pumps	42 (35) 4 (80)^c^	46 (43)	
Style	School or church shoes	35 (29)		
Worn	This type of footwear is commonly worn indoors, or during organised care (i.e. Nursery school or kindergarten)	80 (66)	85 (80)	
Worn	This footwear is commonly worn during special, or more dressy occasions	83 (69)	95 (90)	
Worn	This type of footwear can be worn in variable temperatures and seasons	25 (21)		
Worn	This type of footwear is commonly worn when not being physically active	12 (10)		
Feature	This footwear covers the toes, but does not fully cover the top of the foot, and is secured by a strap at the ankle	93 (77)		
Feature	This footwear commonly has a flat and non-slip sole	51 (42) 13 (50)^b^	86 (81)	
Feature	This footwear has a thin sole with variable flexibility to bending	46 (38)		
Feature	The upper material of the footwear is commonly either made of leather or synthetic materials, which has a rounded shape over the toes	41 (34) 3 (60)^c^	98 (92)	
Feature	The footwear finishes under the ankle	31 (26)		
Feature	The footwear is light weight	4 (3)		
Feature	The footwear has no arch support	5 (4)		
Style	**Boat shoes**	57 (47) *14 (54)*^*b*^	93 (88)	
Style	Slip-ons	36 (30)		
Style	**Loafers**	67 (55)	99 (93)	
Style	Moccasins	28 (23)		
Worn	Worn during warmer weather	39 (32)		
Worn	Boat shoes or loafers are commonly worn during a special or more formal occasion	85 (70)		
Worn	Worn to specific places such as school or church	23 (19)		
Worn	Worn during periods of low physical activity	16 (13)		
Worn	The footwear is for everyday use	4 (3)		
Feature	The uppers of boat shoes or loafers are commonly made of either firm leather or fabric	36 (30) 4 (80)^c^	100 (94)	
Feature	Footwear has a flexible flat sole	51 (42)		
Feature	The footwear upper is commonly soft	42 (35)		
Feature	The footwear covers the heel with minimal heel counter stiffness	37 (31)		
Feature	Boat shoes or loafers are commonly slip on	74 (61)	106 (100)	
Feature	The footwear cuts low under the ankles	32 (26)		
Feature	The footwear is light weight	2 (2)		
Feature	The footwear has variable fixtures	13 (11)		
Style	Thongs, flip flops, slides or jandals	69 (57)	98 (92)	
Worn	Thongs, Flip flops, slides or jandals may be worn in hot weather	ND	100 (94)	
Feature	Thongs, Flip flops, slides or jandals commonly have a flexible sole and are held onto the top of the foot with a strap across the front of the foot only	ND	93 (88)	
Style	Gumboots or Wellingtons	42 (35) 13 (50)^b^	100 (94)	
Worn	Gumboots or Wellingtons are worn in wet weather	ND	98 (92)	
Feature	Gumboots or Wellingtons are made of a waterproof material	ND	97 (92)	
Feature	Gumboots or Wellingtons can easily slip on and off the feet because of their shape and no fasteners	ND	94 (89)	
Style	Slippers	22 (18)		
Style	Crocs	35 (29)		

*Where total responses were <50%, but one or more groups had a 50% or greater response, the additional highest subgroup response and percentage is also provided where a) Health professionals (n = 90), b) Parents (n = 26) and c) Footwear industry (n = 5)

ND–Not displayed–worn and feature questions not displayed and the footwear styles were generated from “other” questions by participants.

Colour Legend: Red Not progress to next round, Orange Progressed to next round, Green Accepted

**Table 4:** Generated statements about how to describe the common features of footwear and round in which the statements were accepted.

Domain	Statement	Round 1 n (%) of 121 participant responses* n (%)^*a-90*, *b-26 or c-5*^	Round 2 (n) % of 105 participant repsonses
Sole flexibility	Flexibility statement 1 when sole is able to twist >45^o^ and bend >45^o^ at the forefoot: The sole should be described as flexible with additional words to convey flexibility to a great extent such as "fully flexible", "extremely flexible" or "very flexible".	86 (71)	
Sole flexibility	Flexibility statement 2 when sole is able to twist 10-45^o^ and bend 10-45^o^ at the forefoot: The sole should be described as flexible with additional words to convey flexibility to a medium extent such as "moderately flexible", "semi-flexible".	94 (78)	
Sole flexibility	Flexibility statement 3 when sole is able to twist <10^o^ and bend <10^o^ at the forefoot: The sole should be described as flexible with additional words to convey amount such as not flexible or non-flexible.	76 (63)	94 (89)
Sole flexibility	Flexibility statement 3 when sole is able to twist <10^o^ and bend <10^o^ at the forefoot: The sole should be described in similar terms to convey its hardness such as: Rigid, Stiff or Solid.	42 (35) 4 (80)^c^	92 (87)
Shoe back	Heel counter	67 (55)	96 (91)
Shoe back	Back of shoe	19 (16)	
Shoe back	Heel cup/heel	28 (26)	
Shoe back flexibility	Flexibility statement 1: Picture showing a heel counter bending towards the sole >45^o^: The amount of movement should be described as flexible with additional words to convey flexibility to a great extent such as "fully flexible" or "very flexible".	55 (45) 3 (60)^c^	97 (92)
Shoe back flexibility	Flexibility statement 1: When heel counter bending towards the sole >45^o^: Soft	27 (22)	
Shoe back flexibility	Flexibility statement 1: When heel counter bending towards the sole >45^o^: Flexible	24 (20)	
Shoe back flexibility	Flexibility statement 2: Picture showing a heel counter bending towards the sole 10-45^o^ The amount of movement should be described as flexible with additional words to convey flexibility to a great extent such as "semi-flexible" or "moderately flexible".	67 (55)	97 (92)
Shoe back flexibility	Flexibility statement 2: Picture showing a heel counter bending towards the sole 10-45^o^ The amount of movement should be described as firm with additional words to convey firmness to a great extent such as "semi-firm" or "moderately firm".	19 (16)	
Shoe back flexibility	Flexibility statement 3: When heel counter is able to bend towards the sole <10^o^ The amount of movement should be described in similar terms to convey its hardness such as: Rigid, Stiff or Solid.	79 (65)	94 (89)
Shoe back flexibility	Flexibility statement 3: When heel counter is able to bend towards the sole <10^o^ The amount of movement should be described in similar terms to convey its limited flexible such as non-flexible or inflexible	23 (19) 3 (60)^c^	93 (88)
Adjustability collective	Fasteners	75 (62)	96 (91)
Adjustability collective	Laces	37 (31)	
Adjustability collective	Straps	28 (23)	
Adjustability collective	Velcro	30 (25)	
Adjustability collective	Buckle	31 (26)	
Adjustability collective	Closures/Fixtures	17 (14)	

*Where total responses were <50%, but one or more groups had a 50% or greater response, the additional highest subgroup response and percentage is also provided where a) Health professionals (n = 90), b) Parents (n = 26) and c) Footwear industry (n = 5)

Colour Legend: Red Not progress to next round, Orange Progressed to next round, Green Accepted

### Agreement

Round 2 took approximately 25 minutes and Round 3 less than 5 minutes for participants to complete. Tables 3 and 4 provide an outline of the statements progressing through Round 2 and Round 3 using the frequency (%) and same colour coding system as Round 1. In Round 2, there were 57 statements with 70% or greater agreement, one statement with 50–59% agreement and one statement discarded. The final statement reached agreement in Round 3. On review of the consensus results, it was acknowledged that participants were presented with one statement in Rounds 2 and 3 that should have been discarded following Round 1, and this been acknowledged in Table 2.

In Round 1, participants provided additional names of other footwear styles. Where there were regional differences in names, these were grouped. For example, flip-flops (United Kingdom and USA), Jandals (New Zealand) and Thongs (Australia) were all considered the same type of shoe. Where consensus was reached on a shoe style or group, the authors generated five new statements of their features and when they would be worn, for presentation to participants in Round 2. In developing these we reviewed the literature and reviewed pictures of the different styles.

**Fig 4:**
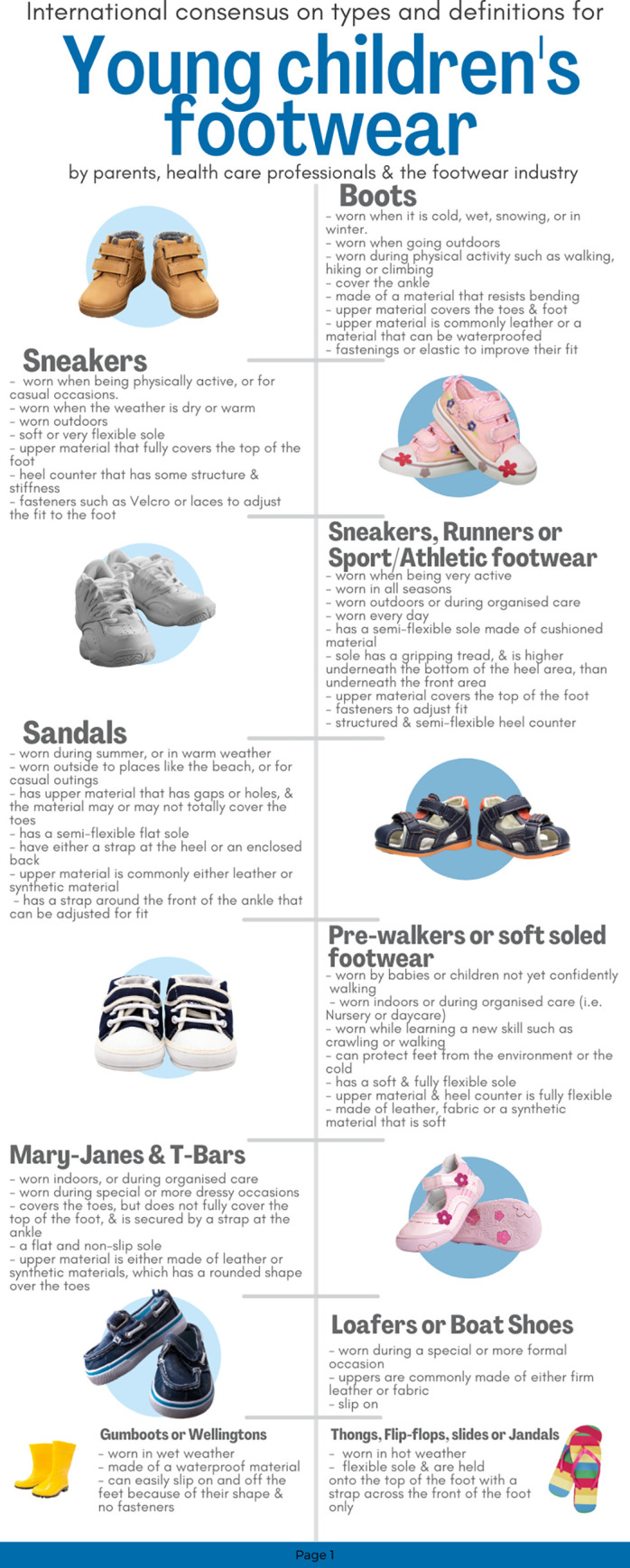
Taxonomy and common features infographic.

**Fig 5:**
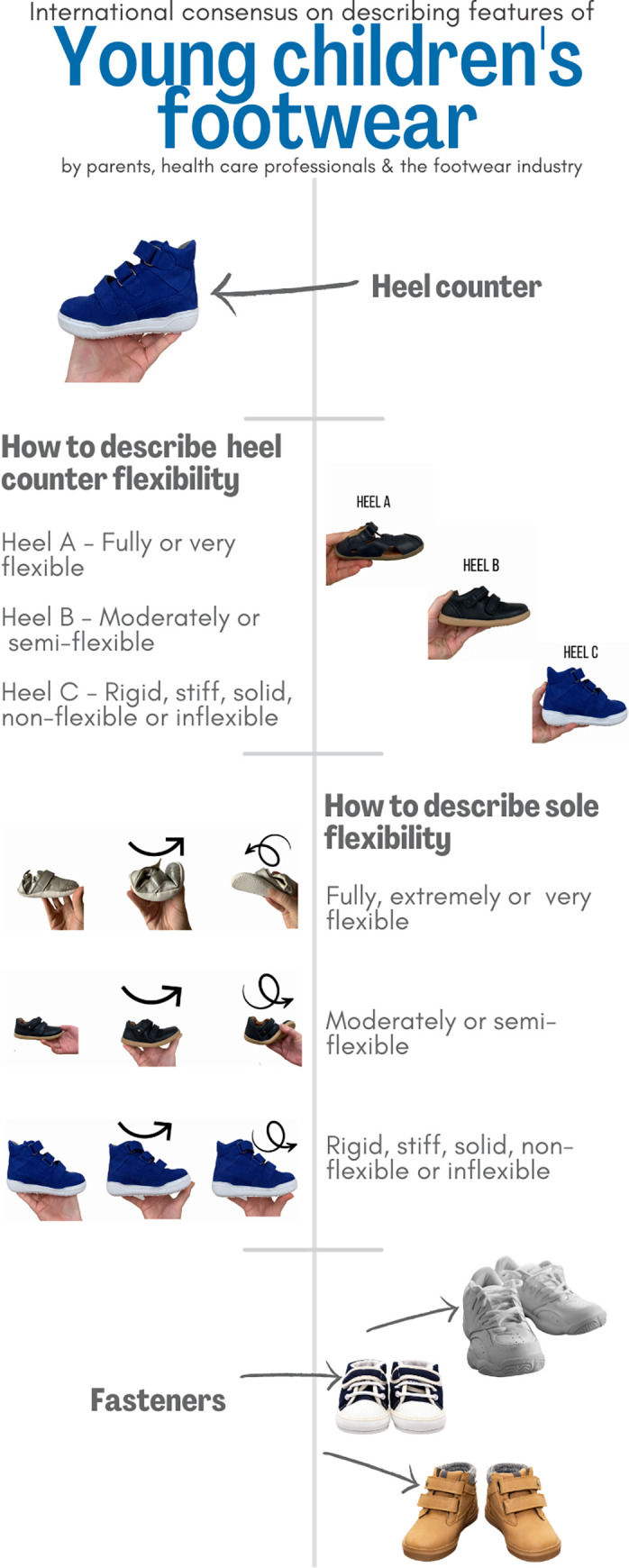
Footwear feature definitions infographic.

The final footwear styles/groupings, when they were commonly worn and the features common to these styles are illustrated in a summary infographic (Figs 4 and 5) using some of the pictures throughout the survey. This infographic also provides details on preferred naming conventions of some parts of the footwear (e.g. heel counter and fasteners) and how participants described the flexibility at different parts of the footwear. For example, a picture showing a heel counter bending towards the sole >45^o^ agreed that the amount of movement should be described as flexible with additional words to convey flexibility to a great extent such as "fully flexible" or "very flexible".

## Discussion

This study offers the first taxonomy for young children’s footwear developed by consensus in consultation with footwear industry professionals, health professionals, and parents. This work was undertaken to respond to persistent challenges with promoting clarity about footwear information, and transparency with footwear research. Emerging from this work, Tables 3 and 4 provide consensus and agreement on footwear styles, such as what features are common in footwear called a boot. Also, when certain types of footwear are commonly worn such as when a boot is worn, and the features common to these types of footwear such as a boot commonly covers the ankle. We have also captured consensus on preferred naming conventions for components of footwear (e.g. heel counter and fasteners) and how the flexibility at different parts of the footwear are described. This taxonomy is a useful resource of contemporary terms and features of footwear, to guide terminology, research and descriptors provided in clinical practice and footwear retail.

Footwear has long been considered a factor impacting on foot development [2, 3] alongside the attainment and improvement of motor skills [16]. Whilst ongoing perspectives about footwear and association with longer-term complications is somewhat controversial, and often omits consideration of the more complex socioeconomic and cultural influences on development [17, 18], it highlights the growing interest in exploring the purpose and function of footwear in childhood and the importance of challenging long-held beliefs. Footwear is an external factor that can influence children’s gait [4, 19], and differences in motor skill [20] meaning that greater consideration of footwear recommendations for toddlers and young children are required. In recent years, the focus on footwear dimensions and fit has been explored [21, 22] but there has also been a shift towards understanding the effects of footwear characteristics on biomechanical outcomes and identifying what features should typify shoes for infants and young children [4, 7]. It is acknowledged that footwear construction is multifactorial and other structural characteristics could influence foot function [8]. The consensus methods used to develop this taxonomy will underpin greater consistency with footwear description and characteristics ensuring clarity of information dissemination in future research, clinical consultations and in marketing. It will enable future researchers to describe a shoe by a term and its common features allowing reproducibility in future footwear research. Further advances in footwear research are needed to offer common understanding of terms, definitions and description of footwear to ensure that research is reproducible and supports the translation of research findings into credible recommendations.

Parents often report concerns about footwear choices for their children [5] and health professionals have an important role providing footwear education and helping parents to navigate information. We believe that the findings from this study are a prerequisite to conversations in practice about footwear choices for children, and it is hoped these findings will assist clinicians with evolving and implementing age-appropriate footwear advice, and helping parents to navigate footwear recommendations. A taxonomy will help health professionals provide accurate descriptions that are acceptable and understood by parents when prescribing footwear with an agreed statement description such as “non-flexible heel counter” or consensus statement “footwear with a moderately flexible sole”. Both features are thought to provide additional stability during developing motor skills [2].

It is important to acknowledge several limitations within this study. The taxonomy was based on consensus opinion, and as manufacturing and patents often are embedded within the design of footwear, terms may differ across countries and footwear sizes. Expert consensus in the context of evidence-based practice constitutes low level evidence. We have attempted to minimise any author bias during this research by co-design with industry, health professionals, and parents in the research team. In acknowledging the Delphi process there is also the limitation with finality and there is no guarantee of correctness [23]. Bias and “group think” of participants was minimised through confidentiality and anonymity during the process. We acknowledge limited participation from Europe and Asia. Engagement in these countries may have influenced the results and researchers are urged to consider how to ensure greater international engagement when undertaking footwear research. Lastly, we did not collect socioeconomic information from parent participants which may play a role in choices about footwear types and the opinions on how these choices impact the child. These factors could have been explored through collection of household income, education level of parent completing the survey and total number of children within the family. Withstanding this, the large number of participants and their diversity played a large role in minimising the impact of localised terms and regional footwear differences. The aim of this study was to develop a taxonomy specific to young children, and as such, generalisability may not be transferable to footwear for older children or adults.

## Conclusion

This taxonomy represents consensus amongst parents, health professionals and footwear designers and retailers, and is an important step in enabling consistency in footwear research. One shoe does not fit all purposes, and the recommendations from this work help to inform the next steps towards ensuring greater transparency and commonality with footwear recommendations. Given the enormity and complexity of the footwear industry, this study underpins the need for further work to explore footwear characteristics and further pursue clear recommendations for parents and shift away from generic recommendations with little validity.

## Supporting information

S1 File Round 1 survey. (DOCX)

S2 File Round 2 survey. (PDF)

S3 File Round 3 survey. (PDF)
