# Supplementary material for: Young children’s footwear taxonomy: An international Delphi survey of parents, health and footwear industry professionals
Source: PLoS One. 2022 Jun 9;17(6):e0269223. doi: 10.1371/journal.pone.0269223 (PMC9182301; doi:10.1371/journal.pone.0269223)
Supplement: S2 File — (PDF) [file pone.0269223.s002.pdf]

INTRO/CONSENT

Round 2

What does my participation involve in this round?

We have collated the responses from the first round.

We had 121 people take part in helping define and describe the characteristics of young children's footwear that you can purchase in shoe stores.

Where greater than 70% of all people who took part said the same thing, a consensus was achieved and we included the name or description, and will present this to you in this survey for your information.

Where greater than 50% of people in any one group responded the same, we have included it as a statement for you to rate how much you agree. Where

less than 50% of people responded the similarly, this has not been included in this round.

There were different groups of people who took part in this research. The groups of people were:
1. Parents of a child or children under the age of 6
2. Young children's footwear sellers, designers or researchers
3. Health Professionals who recommend footwear for children under the age of 6 (some may also have children under the age of 6)

Because there were different amounts of responses between the different groups of people, we didn't want one group's opinion to overpower the opinion of another group. We have presented the results for agreement taking into account the opinions of those in each group.

To meet the aim of this research, it is very important you complete this survey to give us your opinion of the statements. It should take under 10 minutes.

If you would like a copy of your original responses in Round 1, please contact Cylie: cylie.williams@monash.edu

Please provide your email below so we can track responses and link them between groups in each round. Please use your same email for each round.

Boots

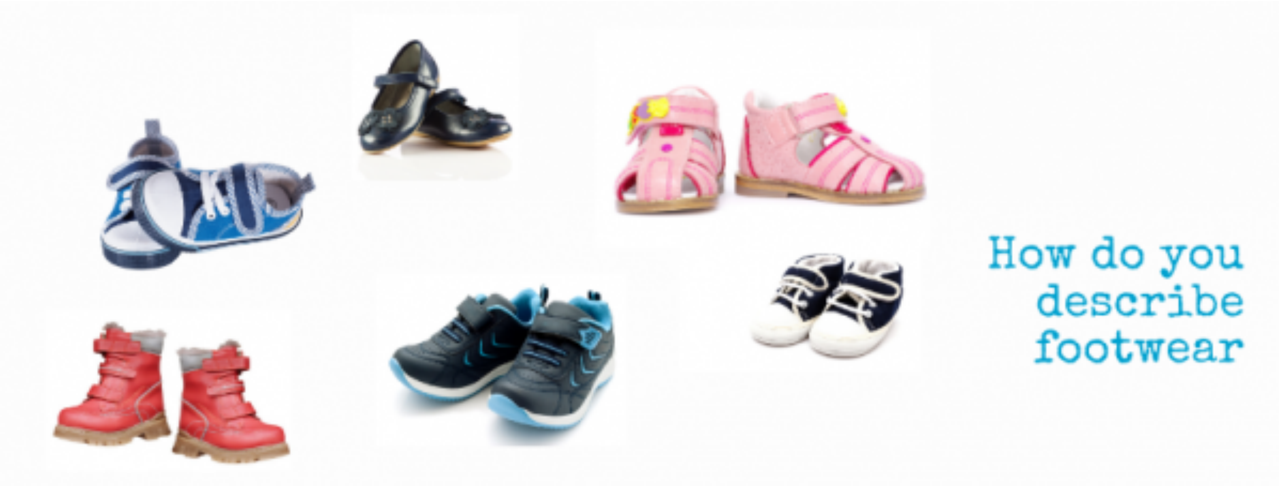

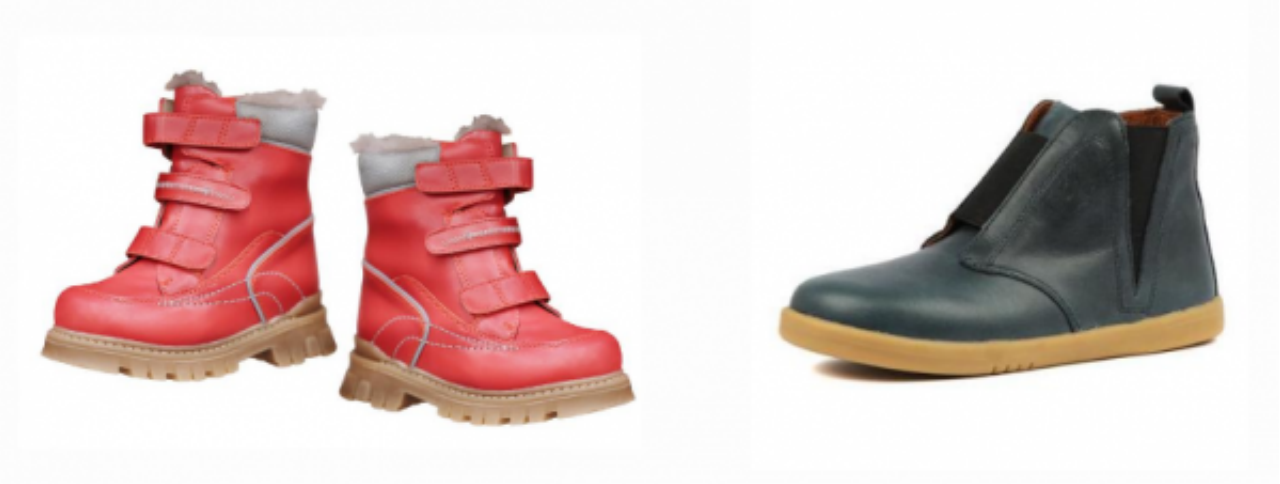

When you were asked about what you called this type of footwear for young children, the name consensus was:

Boots

When you were asked about when young children would wear boots, a consensus was obtained for this statement:

1. Boots are worn when it is cold, wet, snowing, or in winter.

The following reasons or times boot are worn were also identified similarly by greater than 50% in all groups.

Please rate on the following scale whether you agree when boots should be worn.

	Strongly disagree	Disagree	Agree	Strongly Agree
Boots are worn when going outdoors	○	○	○	○
Boots are worn during physical activity such as walking, hiking or climbing	○	○	○	○

If you disagree with any of the above statements, please provide your reason or alternative wording suggestions.

When you were asked about the common features of boots for young children, consensus was obtained for this statement:

1. Boots cover the ankle

The following features of boots were also identified similarly by greater than 50% in all groups.

Please rate on the following scale whether you agree about the common other features of boots:

.

	Strongly disagree	Disagree	Agree	Strongly Agree
The boot sole is commonly made of a material that resists bending	○	○	○	○
The upper material of the boot covers the toes and foot	○	○	○	○
The upper material of boots are leather or material that can be waterproofed	○	○	○	○
Boots are commonly fastened or have elastic to improve their fit.	○	○	○	○

If you disagree with any of the above statements, please provide your reason or alternative wording suggestions.

casual runners

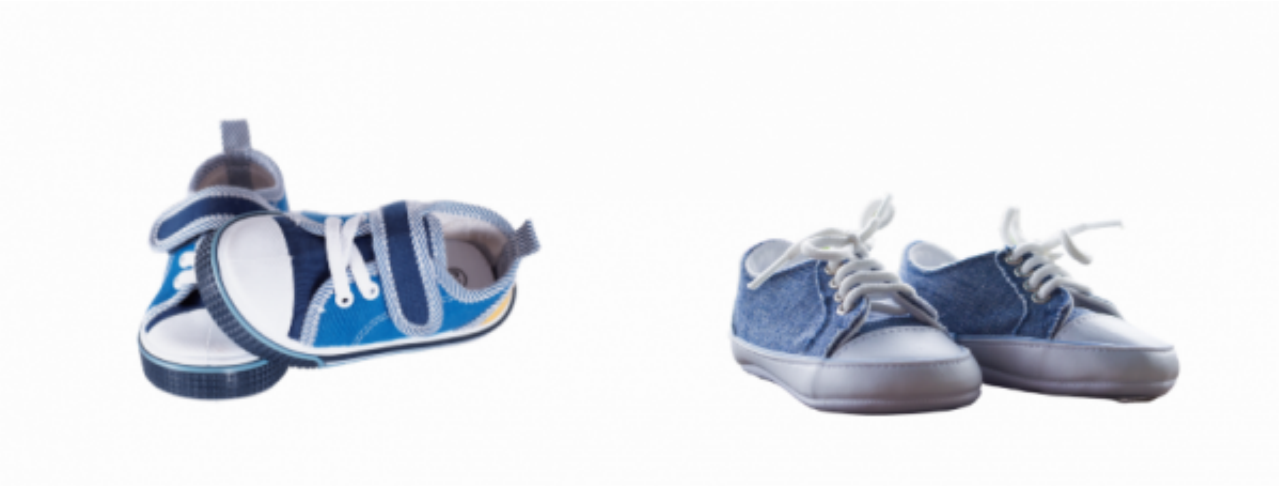

When you were asked about what you called this type of footwear for young children, there was a wide variety of names presented for the footwear.

The following name was identified similarly by greater than 50% in two groups.

Please rate on the following scale whether you agree on this name for this STYLE of footwear:

	Strongly Disagree	Disagree	Agree	Strongly Agree
Sneaker	○	○	○	○

If you disagree with the above name, please provide your reason or alternative wording suggestions.

When you were asked when young children would commonly wear this footwear, consensus was obtained for this statement:

1. The footwear is worn when children are being active, or during casual occasions, including play or other events (e.g. family gatherings).

The following reasons or times this footwear are worn were identified similarly by greater than 50% in all groups.

Please rate on the following scale whether you agree when this type of footwear should be worn.

	Strongly Disagree	Disagree	Agree	Strongly Agree
This footwear is worn when the weather is dry or warm	○	○	○	○
This footwear is worn when outdoors	○	○	○	○

If you disagree with any of the above statements, please provide your reason or alternative wording suggestions.

When you were asked about the common features of this type of footwear for young children, consensus was obtained for two statements:

1. This footwear commonly has a soft or very flexible sole
2. This footwear upper material fully covers the top of the foot

The following features of this footwear were also identified similarly by greater than 50% in all groups

Please rate on the following scale whether you agree about the common other features of this footwear:

	Strongly Disagree	Disagree	Agree	Strongly Agree
This footwear has a heel counter that has some structure and stiffness	○	○	○	○
This footwear has fasteners such as velcro or laces to adjust the fit to the foot	○	○	○	○

If you disagree with any of the above statements, please provide your reason or alternative wording suggestions.

Runners

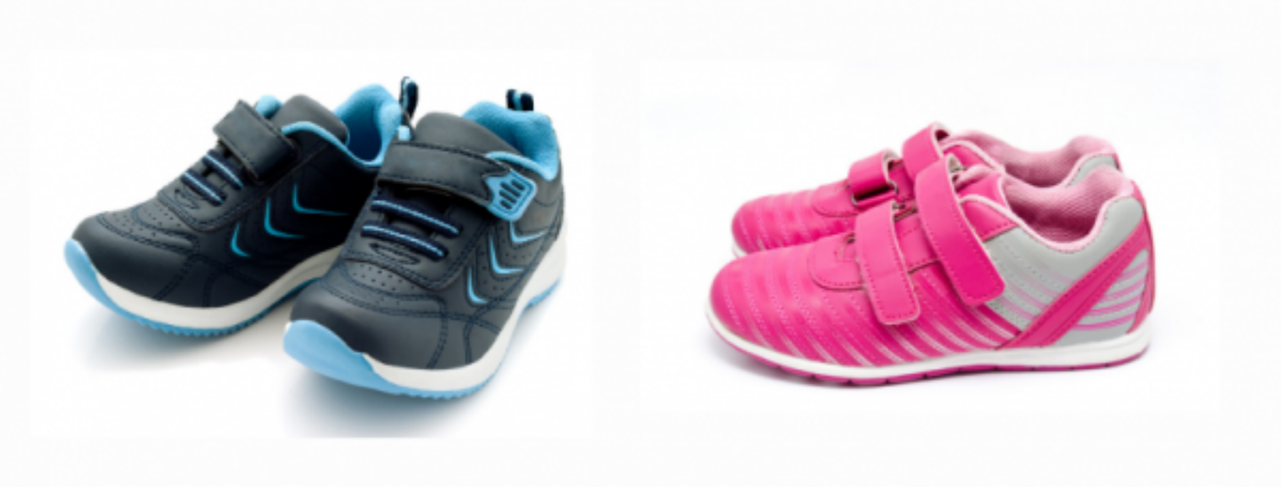

When you were asked about what you called this type of footwear for young children, there was a wide variety of names presented for the footwear.

The following name was identified similarly by greater than 50% in all groups.

Please rate on the following scale whether you agree on these names for this STYLE of footwear (you can agree/disagree with as many as you wish) :

	Strongly Disagree	Disagree	Agree	Strongly Agree
Runners	○	○	○	○
Sport/athletic footwear	○	○	○	○

If you disagree with any of the above names, please provide your reason or alternative wording suggestions.

When you were asked about when young children would wear this footwear, a consensus was obtained for this statement:

1. This footwear is commonly worn when being very active, such as running or playing sport

The following reasons or times this footwear are worn were also identified similarly by greater than 50% in all groups

Please rate on the following scale whether you agree when this footwear should be worn.

Strongly Disagree	Disagree	Agree	Strongly Agree

This footwear can be worn in all seasons	○	○	○	○
This footwear is commonly worn outdoors or during organised care (i.e. Nursery school or kindergarten)	○	○	○	○
This footwear can be worn everyday	○	○	○	○

If you disagree with any of the above statements, please provide your reason or alternative wording suggestions.

When you were asked about the common features of this type of footwear for young children, no consensus was obtained.

The following features of this footwear were also identified similarly by greater than 50% in all groups.

Please rate on the following scale whether you agree about the common other features of this footwear:

	Strongly Disagree	Disagree	Agree	Strongly Agree
The footwear sole resists bending and is made of cushioned materials	○	○	○	○

The bottom of the footwear has a gripping tread, and is higher underneath the bottom of the heel area than underneath the front area	○	○	○	○
The footwear upper material covers the toes and top of the foot	○	○	○	○
The footwear has fasteners to adjust its fit	○	○	○	○
Footwear has a heel counter that has some structure and firmness	○	○	○	○

If you disagree with any of the above statements, please provide your reason or alternative wording suggestions.

Sandals

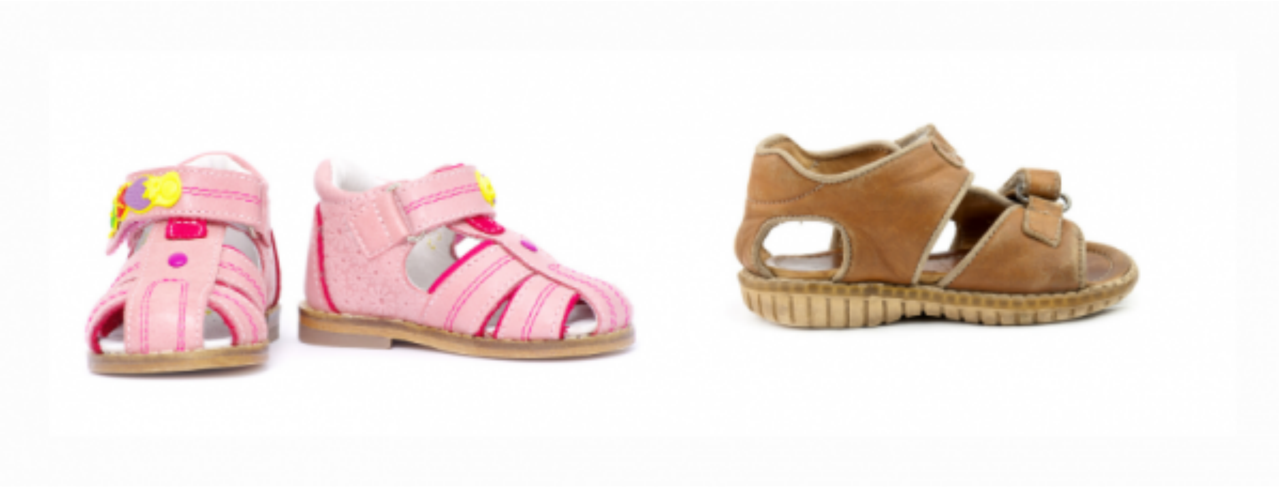

When you were asked about what you called this type of footwear for young children, the name consensus was **Sandals**

When you were asked about when young children would wear sandals, a consensus was obtained for this statement:

1. Sandals are commonly worn during summer, or in warm weather

The following reasons or times this footwear are worn were also identified similarly greater than 50% in all groups.

Please rate on the following scale whether you agree when this footwear should be worn.

	Strongly Disagree	Disagree	Agree	Strongly Agree
Sandals are commonly worn outside to places like the beach, or for casual outings	○	○	○	○

If you disagree with the above statement, please provide your reason or alternative wording suggestions.

When you were asked about the common features of sandals for young children, consensus was obtained for this statement:

1. Sandals commonly have upper material (the top part of the footwear) that has gaps or holes, and the material may or may not totally cover the toes

The following features of this footwear were also identified similarly by greater than 50% in all groups.

Please rate on the following scale whether you agree about the common other features of this footwear:

	Strongly Disagree	Disagree	Agree	Strongly Agree
Sandals have a flat sole with some resistance to bending	○	○	○	○
Sandals can have a strap at the heel or an enclosed back	○	○	○	○
The upper material of the sandal can either be leather or a synthetic material	○	○	○	○

Sandals usually have an ankle strap that can be adjusted for fit	○	○	○	○

If you disagree with the above statements, please provide your reason or alternative wording suggestions.

Mary janes

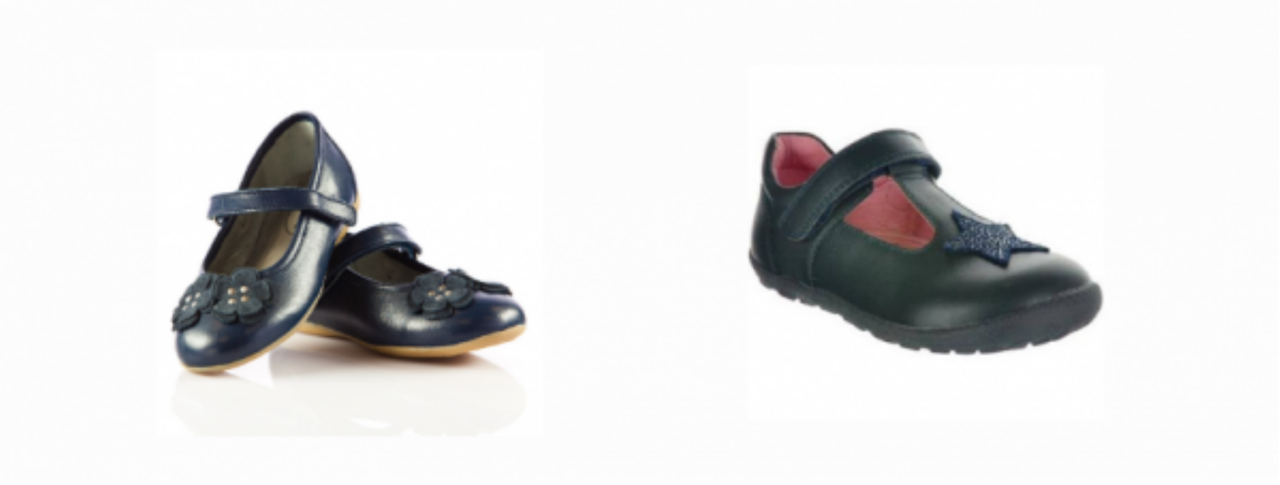

When you were asked about what you called this type of footwear for young children, there was a wide variety of names presented for the footwear.

The following name was identified similarly by greater than 50% in all groups.

Please rate on the following scale whether you agree on these names for this STYLE of footwear:

	Strongly Disagree	Disagree	Agree	Strongly Agree
Mary Janes	○	○	○	○
T-bars	○	○	○	○
Ballet flats	○	○	○	○

If you disagree with the above names, please provide your reason or alternative wording suggestions.

When you were asked about when young children would wear this type of footwear, no consensus was obtained.

The following reasons or times this footwear are worn were also identified similarly by greater than 50% in all groups.

Please rate on the following scale whether you agree when this footwear should be worn.

	Strongly Disagree	Disagree	Agree	Strongly Agree
This footwear is commonly worn indoors or during organised care (i.e. Nursery school or kindergarten)	○	○	○	○
This footwear is commonly worn during special, or more dressy occasions	○	○	○	○

If you disagree with the above statements, please provide your reason or alternative wording suggestions.

When you were asked about the common features of this footwear for young children, consensus was obtained for this statement:

1. This footwear covers the toes but does not fully cover the top of the foot, and is secured by a strap

The following features of this footwear were also identified similarly by greater than 50% in two groups.

Please rate on the following scale whether you agree about the common other features of this footwear:

	Strongly Disagree	Disagree	Agree	Strongly Agree
This footwear has a flat and non-slip sole	○	○	○	○
The upper material of the footwear is either made of leather or synthetic, which has a rounded shape over the toes	○	○	○	○

If you disagree with the above statements, please provide your reason or alternative wording suggestions.

Infant shoes

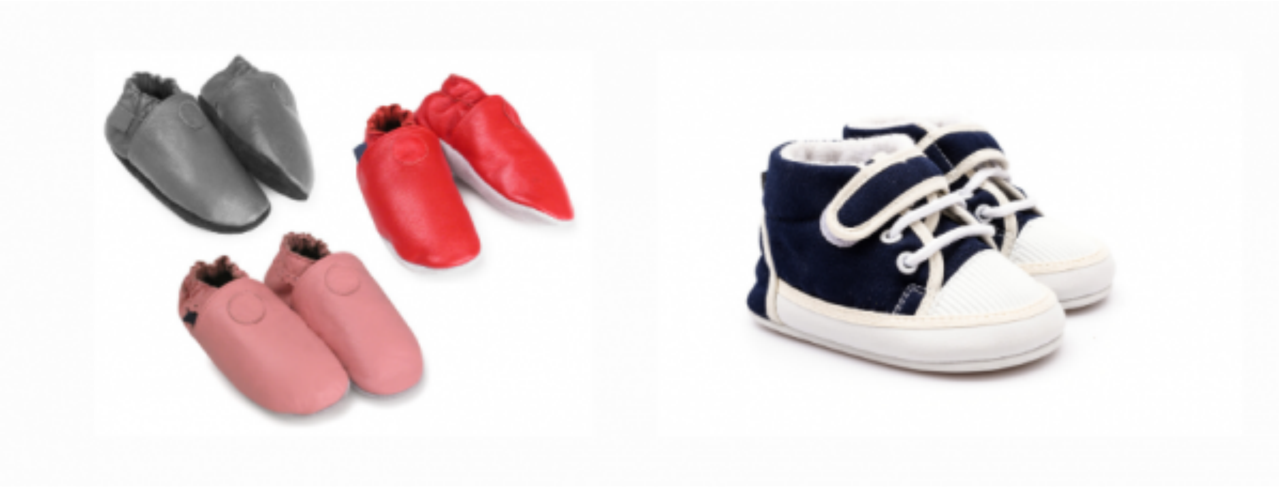

When you were asked about what you called this type of footwear for young children, there was a wide variety of names presented for the footwear.

The following name was identified similarly by greater than 50% in one group.

Please rate on the following scale whether you agree on these names for this STYLE of footwear:

	Strongly Disagree	Disagree	Agree	Strongly Agree
Pre-walker	○	○	○	○
Soft-soled shoes	○	○	○	○

If you disagree with the above names, please provide your reason or alternative wording suggestions.

When you were asked about when young children would wear this type of footwear, no consensus was obtained.

The following reasons or times this footwear are worn were also identified similarly by greater than 50% in all groups.

Please rate on the following scale whether you agree when this footwear should be worn.

	Strongly Disagree	Disagree	Agree	Strongly Agree
This footwear is worn by babies or children not yet confidently walking	○	○	○	○
This footwear is commonly worn indoors or during organised care (i.e. Nursery or daycare)	○	○	○	○
This footwear is commonly worn while learning a new skill such as crawling or walking	○	○	○	○
This footwear protects feet from the environment or the cold	○	○	○	○

If you disagree with the above statements, please provide your reason or alternative wording suggestions.

When you were asked about the common features of this footwear for young children, consensus was obtained for these statements:

1. This footwear has a soft and fully flexible sole
2. The upper material and heel area (heel counter) of this footwear is soft and flexible

The following features of this footwear were also identified similarly by greater than 50% in one group.

Please rate on the following scale whether you agree about the common other features of this footwear:

	Strongly Disagree	Disagree	Agree	Strongly Agree
The upper of the footwear is either made of leather, fabric or a synthetic material that is soft.	○	○	○	○

If you disagree with the above statements, please provide your reason or alternative wording suggestions.

cas

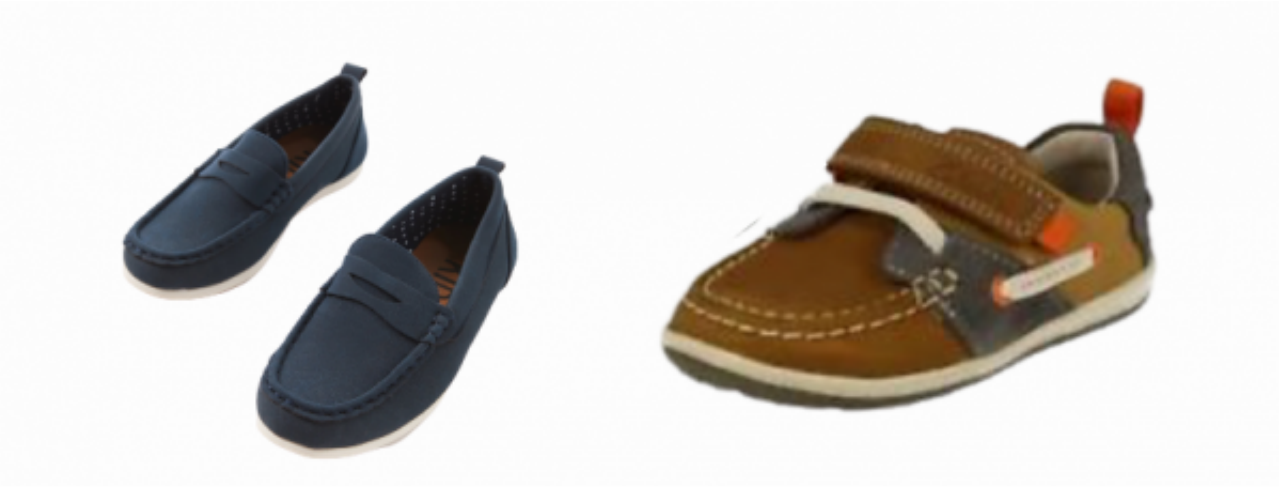

When you were asked about what you called this type of footwear for young children, there was a wide variety of names presented for the footwear.

The following name was identified similarly by greater than 50% in two groups.

Please rate on the following scale whether you agree on these names for this STYLE of footwear:

	Strongly Disagree	Disagree	Agree	Strongly Agree
Loafer	○	○	○	○
Boat shoe	○	○	○	○

If you disagree with the above comments, please provide your reason or alternative wording suggestions.

When you were asked about when young children would wear this type of footwear, consensus was obtained for this statement

1. This footwear is commonly worn during a special or more formal occasion

There were no other statements that reached greater than 50% in agreement among each group.

When you were asked about the common features of this footwear for young children, no consensus was made.

The following features of this footwear were also identified similarly by >50% of all groups.

Please rate on the following scale whether you agree about the common other features of this footwear:

	Strongly Disagree	Disagree	Agree	Strongly Agree
The footwear upper is made of either firm leather or fabric	○	○	○	○
This footwear is commonly slip on	○	○	○	○

If you disagree with the above statements, please provide your reason or alternative wording suggestions.

Others shoes

You were asked about any alternative footwear that young children (under the age of 6)

commonly wear that hasn't been featured.

The following type and feature were identified similarly by greater than 50% in health professionals or parents.

Please rate your agreement with the following types/names of footwear that may be worn by young children. Please do not rate it based on suitability, but if you agree this is a type of shoe that young children may wear in certain circumstances or times.

	Strongly Disagree	Disagree	Agree	Strongly agree
Thongs/Flip flops/slides or jandles	○	○	○	○
Gumboots/Wellingtons	○	○	○	○

Please rate your agreement on the following descriptions for the above groups of footwear that may be worn by young children

	Strongly Disagree	Disagree	Agree	Strongly Agree
Thongs/Flip flops/slides or jandles may be worn in hot weather	○	○	○	○
Thongs/Flip flops/slides or jandles commonly have a flexible sole and are held onto the top of the foot with a strap across the front of the foot only	○	○	○	○

Gumboots/Wellingtons are worn in wet weather	O	O	O	O
Gumboots/Wellingtons are made of a waterproof material	O	O	O	O
Gumboots/Wellingtons can easily slip on and off the feet because of their shape and no fasteners	O	O	O	O

Intro to features.

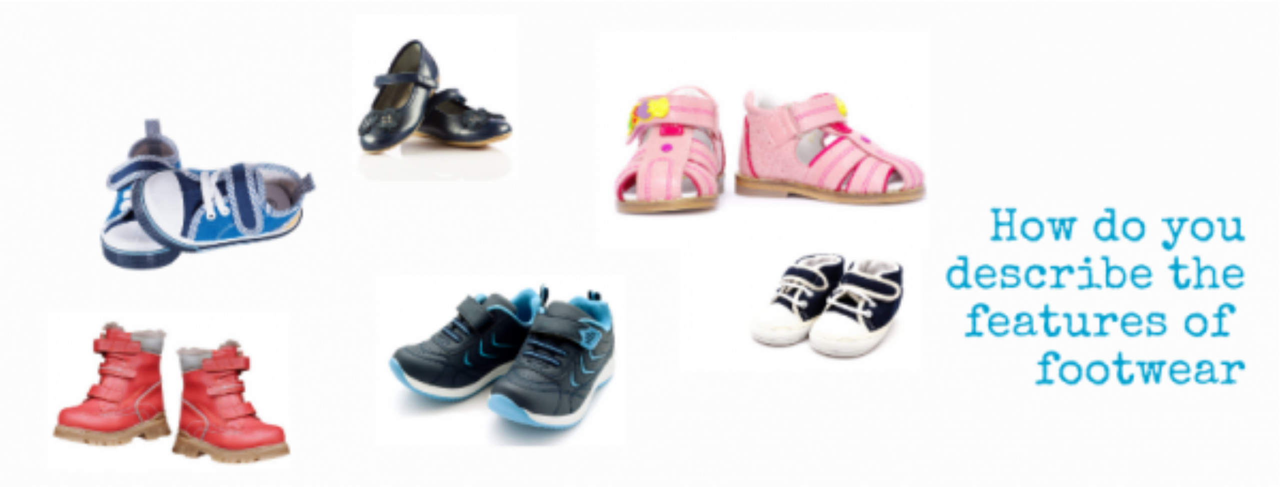

Keep going, you're almost there!

Flexibility features

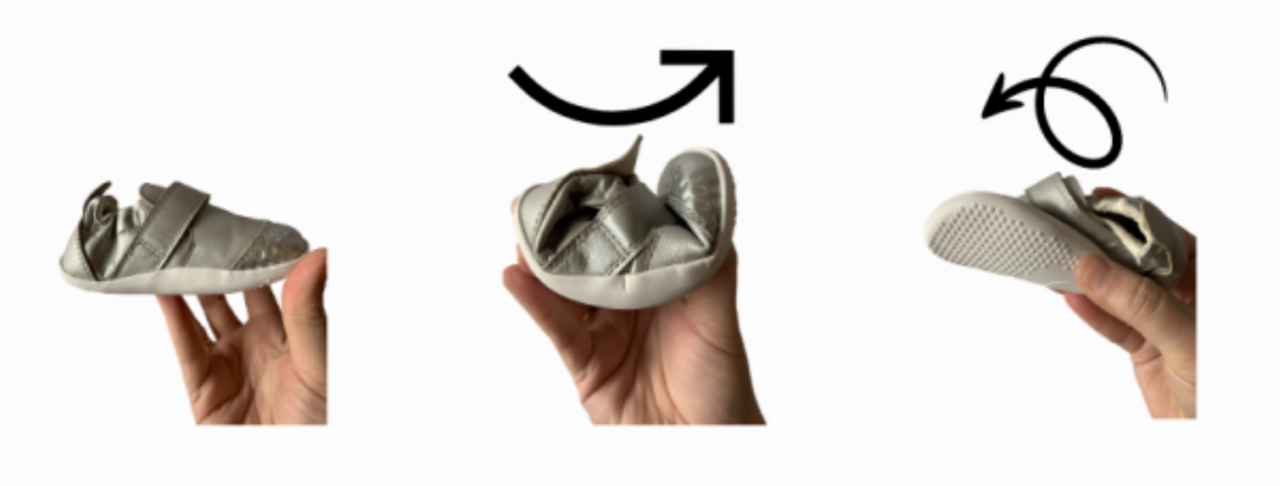

When you were asked about the amount of movement of the sole of the footwear for young children, consensus was obtained:

1. The sole should be described as flexible with additional words to convey flexibility to a great extent such as "fully flexible", "extremely flexible" or "very flexible".

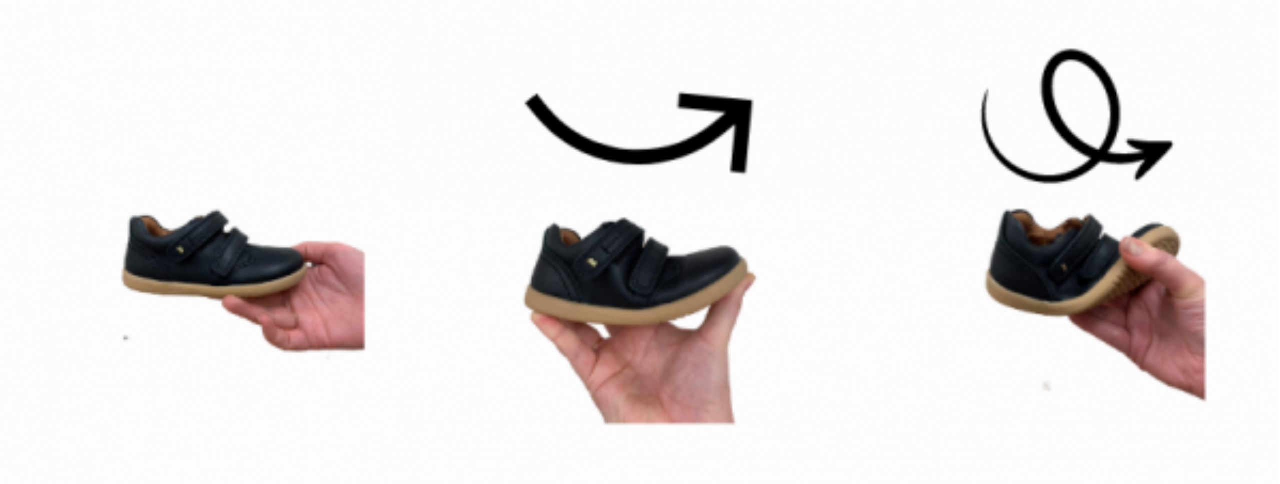

When you were asked about the amount of movement of the sole of the footwear for young children, consensus was obtained:

1. The sole should be described as flexible with additional words to convey

flexibility to a medium extent such as "moderately flexible", "semi-flexible".

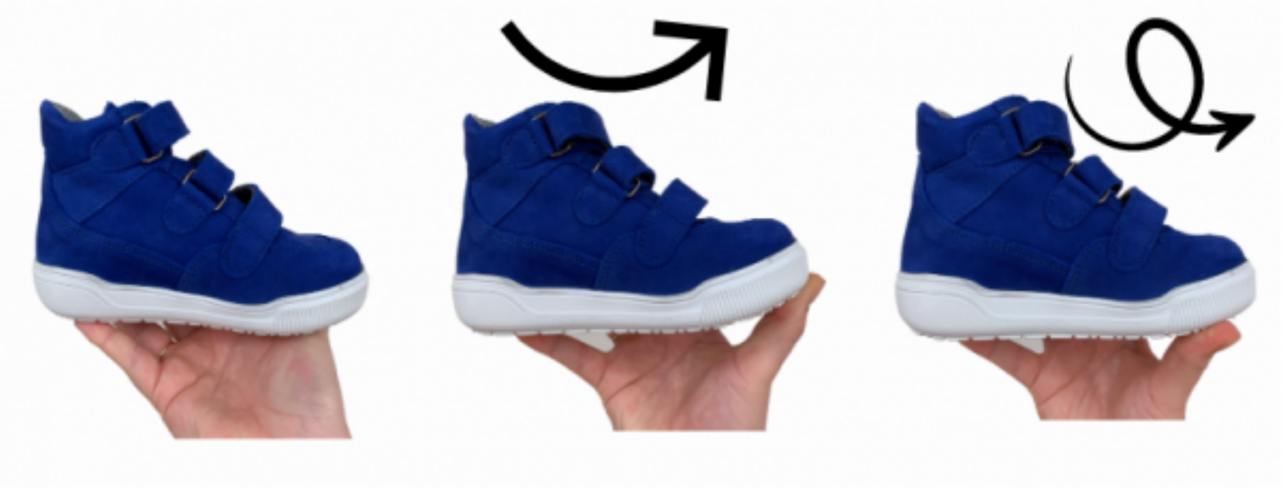

When you were asked about the amount of movement of the sole of the footwear for young children, this was described similarly by greater than 50% in two groups.

Please rate on the following scale whether you agree with this description of the sole bend and twist:

	Strongly Disagree	Disagree	Agree	Strongly Agree
The sole should be described as flexible with additional words to convey amount such as not flexible or non-flexible.	○	○	○	○
The sole should be described in similar terms to convey its hardness such as: Rigid, Stiff or Solid.	○	○	○	○

If you disagree with any of the above statements, please provide your reason or alternative wording suggestions.

Heel counter

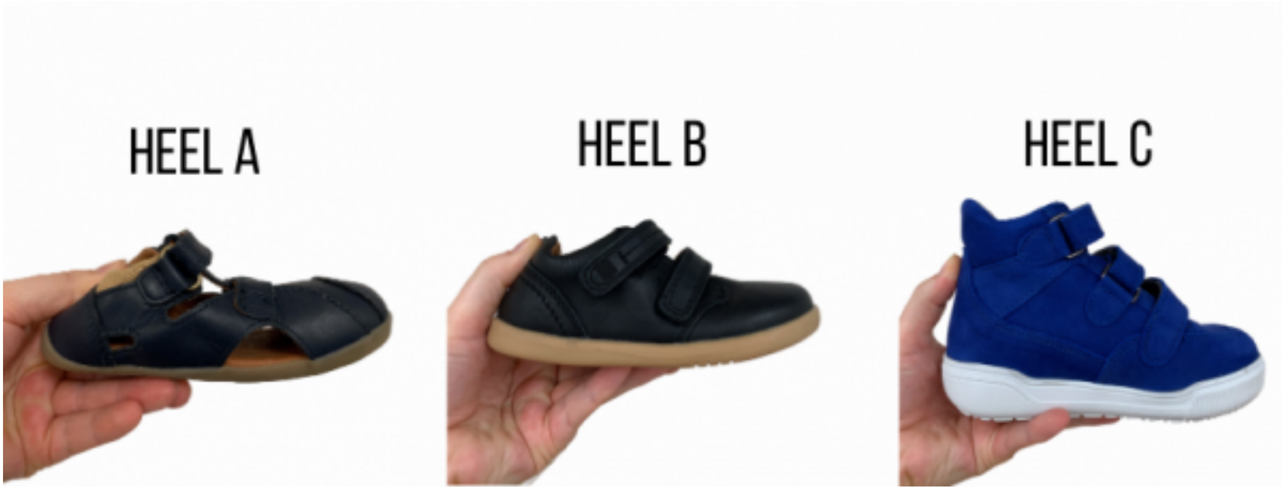

Above is a picture of force being applied to the back of three different shoes.

You were firstly asked what you would describe the back part of the footwear where the thumb was applying the force.

This was described by 50% of two groups.

Please rate on the following scale whether you agree with this term:

	Strongly Disagree	Disagree	Agree	Strongly Agree
Heel counter	○	○	○	○

You were asked how you would describe the movement at HEEL A

This was described similarly by greater than 50% in two groups.

Please rate on the following scale whether you agree with this term:

	Strongly Disagree	Disagree	Agree	Strongly Agree
The amount of movement should be described as flexible with additional words to convey flexibility to a great extent such as "fully flexible" or "very flexible".	○	○	○	○

You were asked how you would describe the movement at HEEL B

This was described similarly by greater than 50% in two groups.

Please rate on the following scale whether you agree with this term:

	Strongly Disagree	Disagree	Agree	Strongly Agree
The amount of movement should be described as flexible with additional words to convey flexibility to a great extent such as "semi-flexible" or "moderately flexible".	○	○	○	○

You were asked how you would describe the movement at HEEL C

This was described similarly by greater than 50% in two groups.

Please rate on the following scale whether you agree with either or both terms:

	Strongly Disagree	Disagree	Agree	Strongly Agree
The amount of movement should be described in similar terms to convey its hardness such as: Rigid, Stiff or Solid.	○	○	○	○
The amount of movement should be described in similar terms to convey its limited flexible such as non-flexible or inflexible	○	○	○	○

If you disagree with any of the above statements, please provide your reason or alternative wording suggestions.

Fasteners

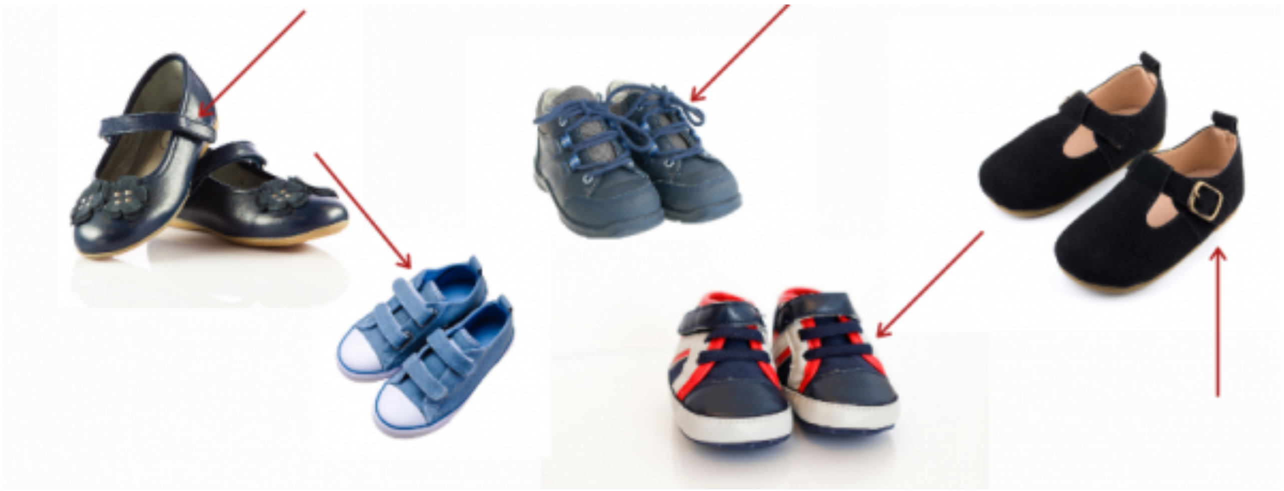

You were asked how you would describe the things identified by the arrows.

This term was used by greater than 50% in all groups.

Please rate on the following scale whether you agree with this term:

	Strongly Disagree	Disagree	Agree	Strongly Agree
Fasteners	○	○	○	○

If you disagree with the above statement, please provide your reason or alternative wording suggestions.

End

Thank you for your responses. We will contact you via email in 4 weeks from the close of this survey. The next survey will ask you rate any statements that are again agreed to by at least 50% of one of more groups. The next survey

will likely be very brief and take less than 5 minutes.

It is important to meet the aims of this research that you keep taking part in each round, so we thank you for your continued involvement.

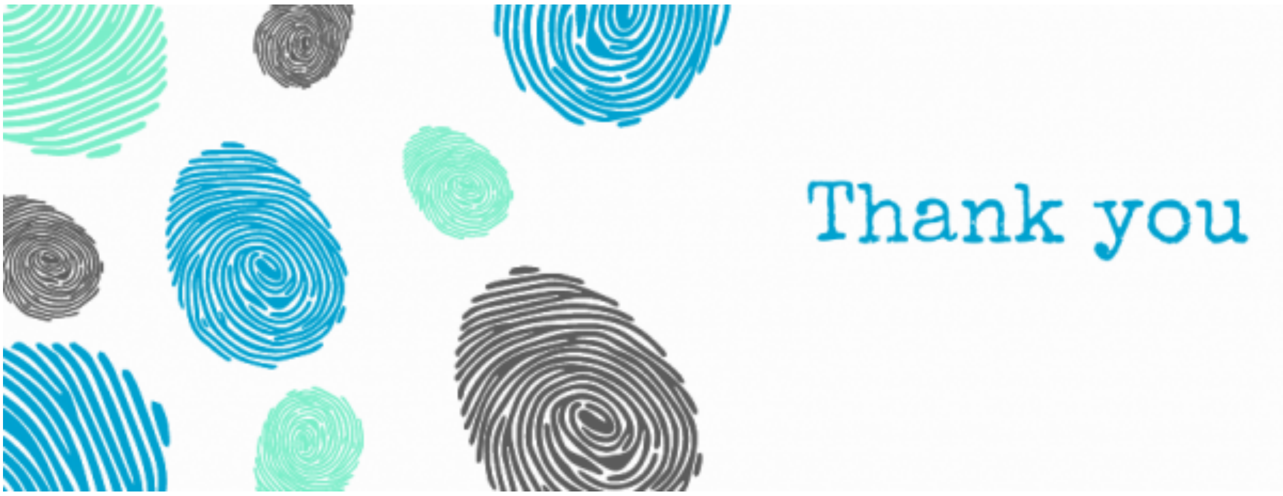

Powered by Qualtrics
