# Supplementary material for: Young children’s footwear taxonomy: An international Delphi survey of parents, health and footwear industry professionals
Source: PLoS One. 2022 Jun 9;17(6):e0269223. doi: 10.1371/journal.pone.0269223 (PMC9182301; doi:10.1371/journal.pone.0269223)
Supplement: S3 File — (PDF) [file pone.0269223.s003.pdf]

INTRO/CONSENT

Round 3

What does my participation involve in this round?

<u>You only have 2 statements to rate your agreement in this last round, it should take less than 5 minutes.</u>

In this round, we also have removed pictures of footwear, because we want you to now concentrate on the descriptions.

If you would like a copy of your original responses from Round 2, please contact Cylie: cylie.williams@monash.edu

Please provide your email below so we can track responses and link them between groups in each round.
Please use your same email for each round.

Runners

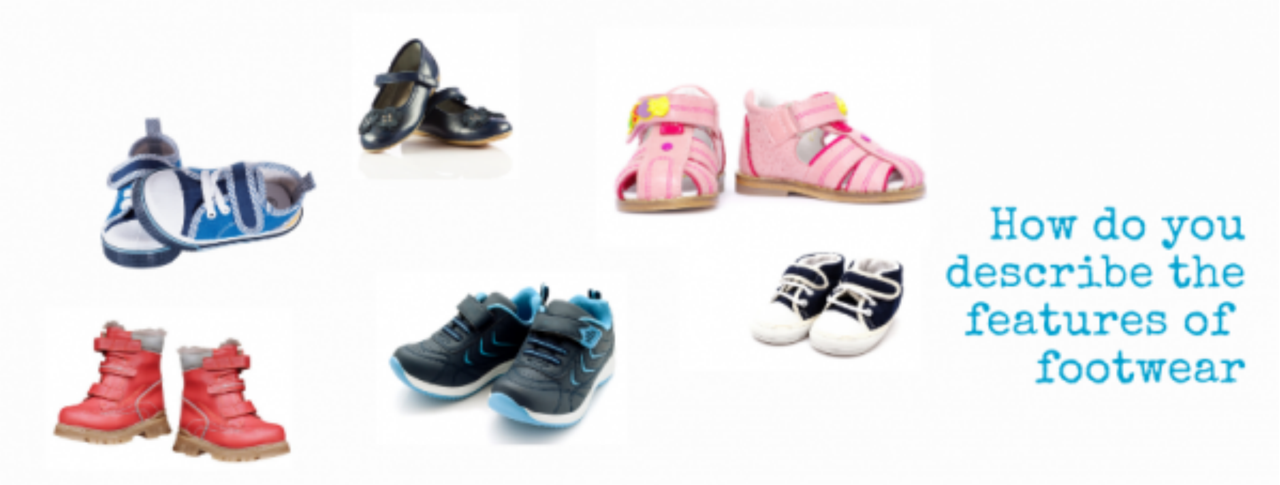

In the first round, you told us various names for a group or type of footwear. Some of these terms were very local to the country or region that you live. There were other terms that people used, however no other term was rated >50%.

And in Round 2, 76-92% of people agreed a type of athletic footwear could be called a **Runner** or **Sports/athletic footwear**

Another name was agreed by greater than 50% of you as a term for footwear that shares some similar features with a **Runner** or **Sports/athletic footwear.** Regardless of if you use this name for the footwear, please rate your agreement with it being one of the names that may be used for this type of footwear, particularly if it may be used in other countries than your own.

	Strongly Disagree	Disagree	Agree	Strongly Agree
**Joggers**	○	○	○	○

When you were asked about when young children would wear this group of similar footwear, a consensus was obtained for this statement:

1. This type of footwear is commonly worn when being very active, such as running or playing sport

And in Round 2, 92%-94% of you also agreed that:

2. This footwear is commonly worn in all seasons
3. This footwear is commonly worn outdoors or during organised care (i.e. Nursery school or kindergarten)
4. This footwear can be worn everyday

And in Round 2, 75%-95% of you also agreed the following statements about this group of footwear:

1. It a semi-flexible sole made of cushioned material
2. The bottom or sole of the footwear has a gripping tread, and is higher underneath the bottom of the heel area than underneath the front area
3. Its upper material covers the top of the foot
4. It has fasteners to adjust fit
5. It commonly has a structured and semi-flexible heel counter

Mary janes

In the first round, you told us various names for footwear that shared some features. Some of these terms were very local to the country or region that you live. There were other terms that people used, however no other term was rated >50%.

And in Round 2, 81% of people agreed one of these types of footwear could be called a Mary-Jane

One name received less than 50% of agreement and has been removed.

Another name was agreed by greater than 50% of you as a term for footwear that shares some similar features to **Mary-Janes**. Regardless of if you use this name for the footwear, please rate your agreement with it being one of the names that may be used for this type of footwear, particularly if it may be used in other countries than your own.

	Strongly Disagree	Disagree	Agree	Strongly Agree
**T-bars**	○	○	○	○

And in Round 2, 80-90% of people agreed on the statements relating to when this footwear is worn:

1. This types of footwear is commonly worn indoors, or during organised care (i.e. Nursery school or kindergarten)
2. This footwear is commonly worn during special, or more dressy occasions

You described the features of this type of footwear in Round 1 as:

1.This footwear covers the toes, but does not fully cover the top of the foot, and is secured by a strap at the ankle

And in Round 2, 81%-92% of you also agreed that:
2. This footwear commonly has a flat and non-slip sole
3. The upper material of the footwear is commonly either made of leather or synthetic materials, which has a rounded shape over the toes

ALL

The following is for your information and the most up to date results of all the footwear names, when they are commonly worn and their common features.

You can read or skip past these to submit your ratings.
Please click on submit at the bottom of the page to submit.

In the first round, you told us boots were a type of footwear commonly worn by young children.
You told us in Round 1:
1. **Boots** are often worn when it is cold, wet, snowing, or in winter.

And in Round 2, 91% of people agreed that:
2. **Boots** are often worn when going outdoors
3. **Boots** are often worn during physical activity such as walking, hiking or climbing

You described the features of boots in Round 1 as:
1. **Boots** cover the ankle

And in Round 2, 79%-94% of you also agreed that:

2. The **boot** sole is commonly made of a material that resists bending
3. The **boot** upper material covers the toes and foot
4. The **boot** upper material is commonly leather or a material that can be waterproofed
5. **Boots** commonly have fastenings or elastic to improve their fit

In the first round, you told us various names for casual footwear. Some of these were very local to the country or region that you live.

And in Round 2, 89-91% of people agreed this type of footwear could be called a Sneaker.

In Round 1, you told us:

1. **Sneakers** are commonly worn when being physically active, or for casual occasions. These activities or occasions may include play, or event based occasions (e.g. family gatherings).

In Round 2, 82%-88% you also agreed that:

2. **Sneakers** are commonly worn when the weather is dry or warm
3. **Sneakers** are commonly worn outdoors

You described the features of sneakers in Round 1 as:

1. **Sneakers** commonly have a soft or very flexible sole
2. **Sneakers** commonly have an upper material fully covers the top of the foot

And in Round 2, 71%-93% you also agreed that

3. **Sneakers** commonly have a heel counter that has some structure and stiffness
4. **Sneakers** commonly have fasteners such as velcro or laces to adjust the fit to the foot

When you were asked about what you called this type of footwear for young children, the name consensus was **Sandals**

In the first round, you told us sandals were a type of footwear commonly worn by young children.
You told us in Round 1:
1. **Sandals** are commonly worn during summer, or in warm weather

And in Round 2, 92% of people agreed that:
2. **Sandals** are commonly worn outside to places like the beach, or for casual outings

You described the features of sandals in Round 1 as:
1. **Sandals** commonly have upper material that has gaps or holes, and the material may or may not totally cover the toes.

And in Round 2, 83%-95% of you also agreed that:
2. **Sandals** commonly have a semi flexible flat sole
3. **Sandals** can have either a strap at the heel or an enclosed back
4. The upper material of **sandals** are commonly either leather or synthetic material
5. **Sandals** commonly have a strap around the front of the ankle that can be adjusted for fit

In the first round, you told us various names for another type of footwear.
And in Round 2, 89%-91% of people agreed that type of footwear could be called pre-walkers or soft-soled footwear

In Round 2, you provided feedback on agreement with statements, however you also told us that these footwear types are commonly worn less often by young children. The statements you agreed on have been modified to "can be worn" rather than "commonly worn" to reflect your feedback.

In Round 2 78%-92% you agreed that:

1. **Pre-walkers** or **soft-soled footwear** can be worn by babies or children not yet confidently walking
2. **Pre-walkers** or **soft-soled footwear** can be worn indoors or during organised care (i.e. Nursery or daycare)
3. **Pre-walkers** or **soft-soled footwear can be** worn while learning a new skill such as crawling or walking
4. **Pre-walkers** or **soft-soled footwear** can protect feet from the environment or the cold

You described the features of this footwear in Round 1 as:

1. **Pre-walker** or **soft-soled footwear** has a soft and fully flexible sole
2. The upper material and heel area (heel counter) of **pre-walkers** or **soft-soled footwear** are fully flexible

And in Round 2, 95% you also agreed that

3. The upper of **pre-walkers** or **soft-soled footwear** is either made of leather, fabric or a synthetic material that is soft.

In the first round, you told us various names for another type of casual footwear.

Round 2, 88%-93% of you agreed this footwear is commonly called boat shoes or loafers

In Round 1, you described when this footwear would be worn:

1. **Boat shoes** or **loafers** are commonly worn during a special or more formal occasion

In Round 2, 90%-95% of you described the common features of this footwear:

1. The uppers of **Boat shoes** or **loafers** are commonly made of either firm leather or fabric
2. **Boat shoes** or **loafers** are commonly slip on

In the first round, you told us various types of other footwear young children commonly wore.

And in Round 2, 92%-94% of people agreed that **thongs, flip flops, slides or jandles** and **gumboots or wellingtons** were also common footwear types.

In Round 2, 88%-94% of you agreed on the following common features of these groups of footwear:

1. **Thongs, Flip flops, slides** or **jandles** may be worn in hot weather
2. **Thongs, Flip flops, slides** or **jandles** commonly have a flexible sole and are held onto the top of the foot with a strap across the front of the foot only
3. **Gumboots** or **Wellingtons** are worn in wet weather
4. **Gumboots** or **Wellingtons** are made of a waterproof material
5. **Gumboots** or **Wellingtons** can easily slip on and off the feet because of their shape and no fasteners

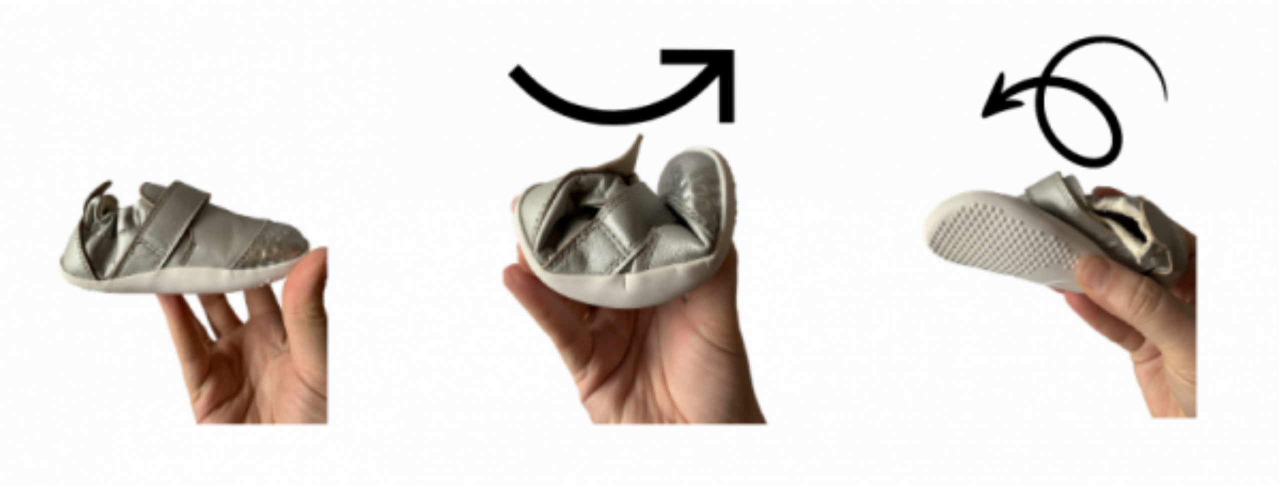

When you were asked about the amount of movement of the sole of the footwear for young children, consensus was obtained:

1. The sole should be described as flexible with additional words to convey flexibility to a great extent such as "fully flexible", "extremely flexible" or "very flexible".

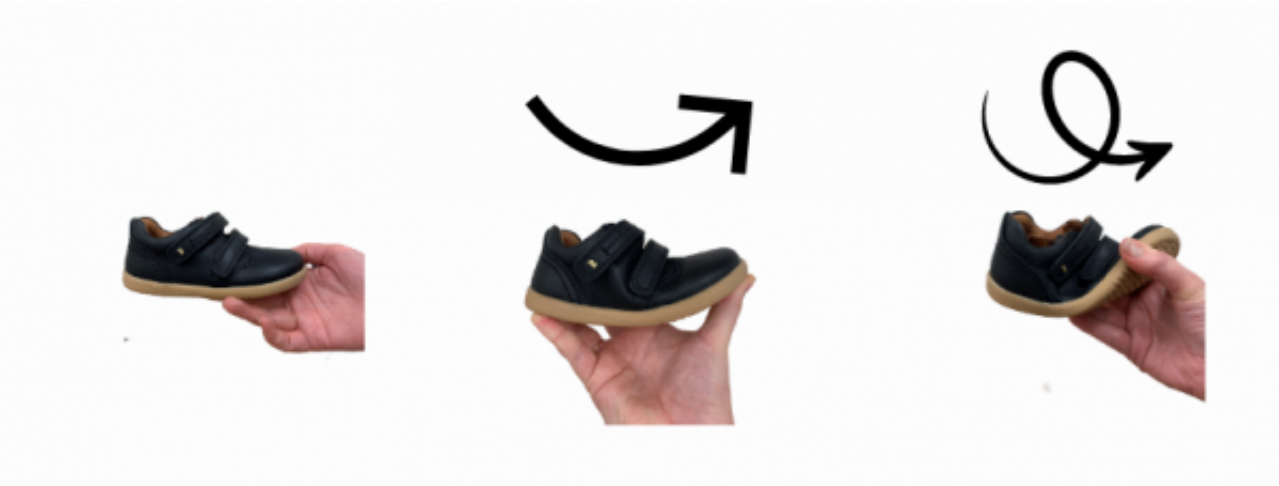

When you were asked about the amount of movement of the sole of the footwear for young children, consensus was obtained:

1. The sole should be described as flexible with additional words to convey flexibility to a medium extent such as "moderately flexible", "semi-flexible".

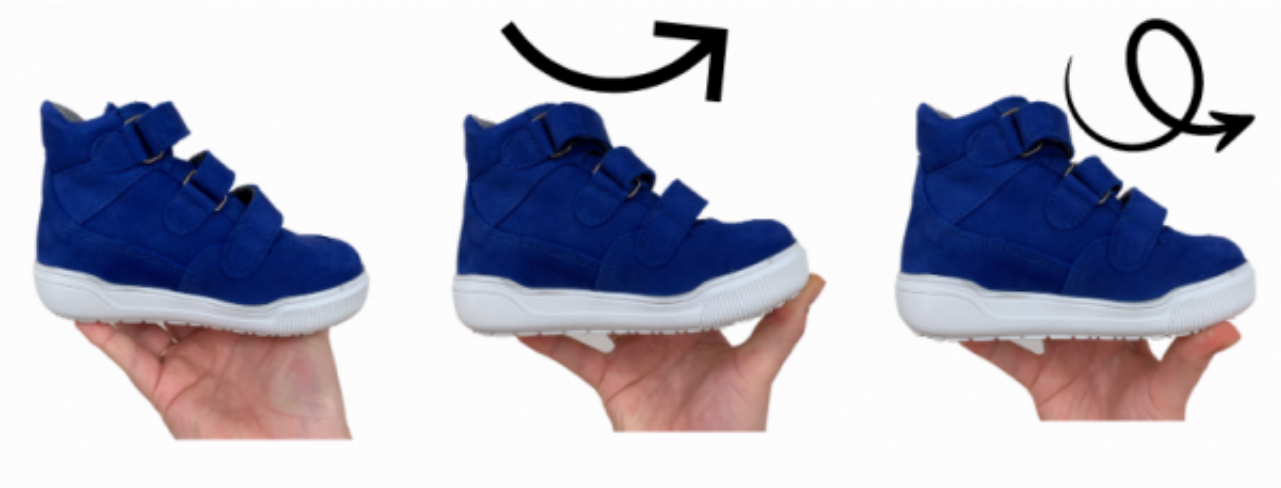

In Round 2, 87-89% of you agreed that:
The sole could be described as flexible, with additional words to convey the amount of flexibility such as not flexible or non-flexible

OR

The sole could be described in similar terms to convey hardness such as: Rigid, stiff or solid.

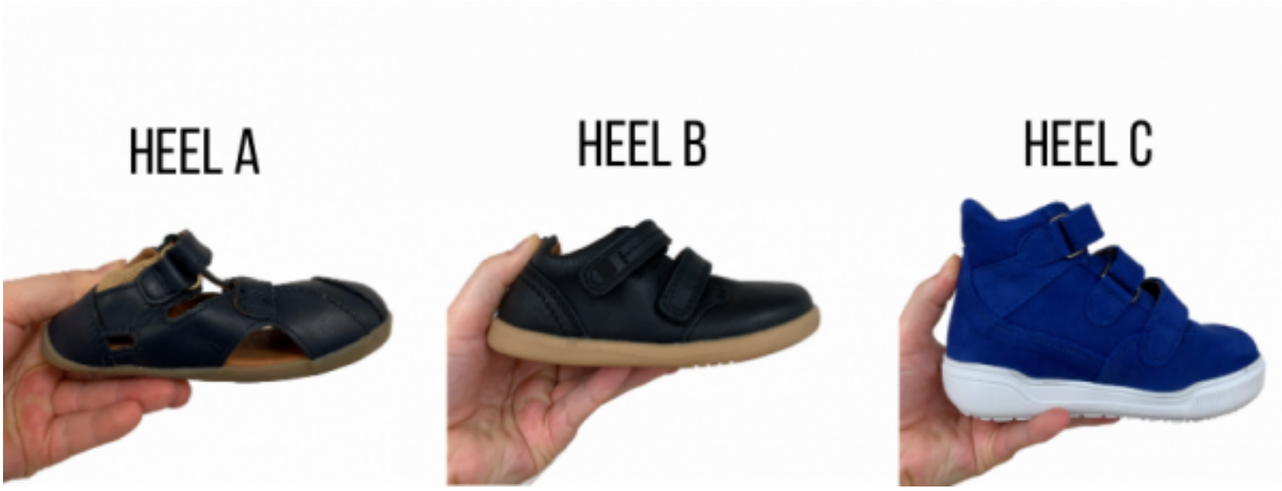

Above is a picture of force being applied to the back of three different shoes.

In Round 2, 91% of you agreed the back of the shoe can be called a heel counter.

In **Round 2**, 92% of you agreed that the amount of movement in **HEEL A** should be described as flexible with additional words to convey flexibility to a great extent such as "fully flexible" or "very flexible".

In **Round 2**, 92% of you agreed that the amount of movement in **HEEL B** should be described as flexible with additional words to convey flexibility to a great extent such as "semi-flexible" or "moderately flexible".

In **Round 2**, 88%-89% of you agreed that **HEEL C** could be described in two ways. Either:

1. The amount of movement should be described in similar terms to convey its hardness such as: Rigid, Stiff or Solid.
2. The amount of movement should be described in similar terms to convey its limited flexible such as non-flexible or inflexible

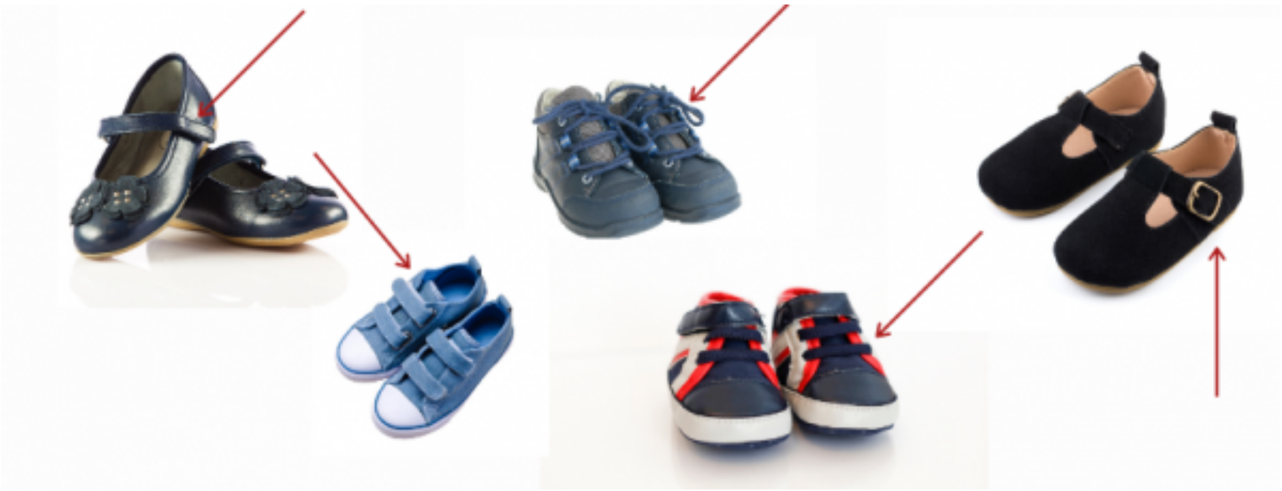

In Round 2, 91% of you agreed that **Fasteners** should be used to describe adjustable features of footwear.

Thank you for your responses. We appreciate your time and support in being part of this research.
Any item that does not reach greater than 70% of agreement in this round will not be included. We will provide you with an outcome statement at the end of

4 weeks or when all 106 participants have responded.

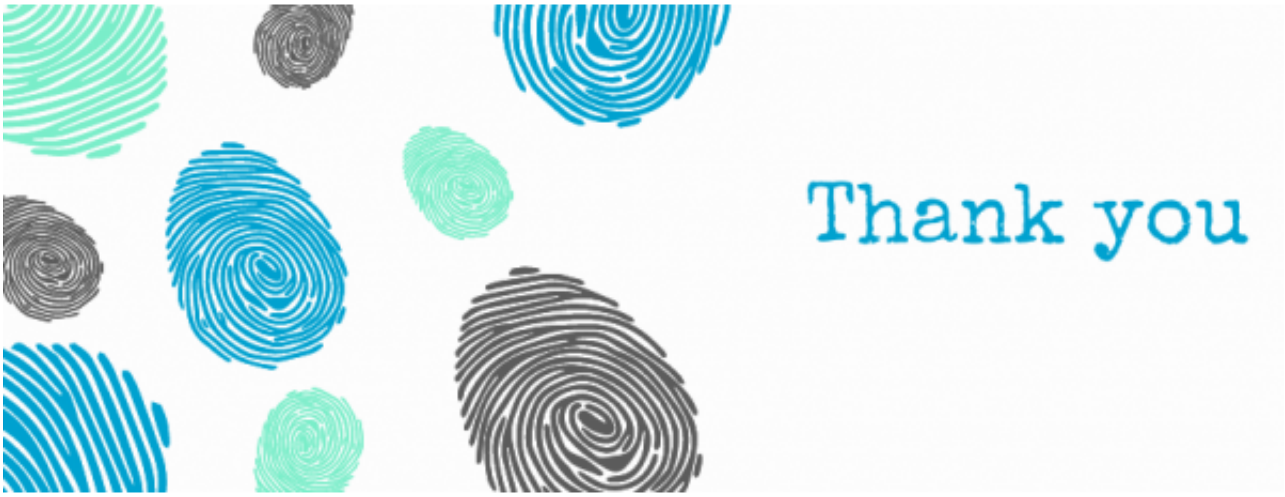

Powered by Qualtrics
