# Supplementary material for: Young children’s footwear taxonomy: An international Delphi survey of parents, health and footwear industry professionals
Source: PLoS One. 2022 Jun 9;17(6):e0269223. doi: 10.1371/journal.pone.0269223 (PMC9182301; doi:10.1371/journal.pone.0269223)
Supplement: Attachment — Submitted filename: Commnents_25_July_2021.docx [file pone.0269223.s004.docx]

**Young children’s footwear taxonomy: an international Delphi survey of parents, health and footwear professionals**

**Abstract:** without comments

**Introduction**: without comments

**Methods:**

Round 1: I consider that is necessary that authors explain how the questions of the initial survey was designed. Are the validated questions? Equally, it is necessary that be explained how was chosen the models of shoes whose were presented to participants

**Results:**

This apart is clear, only the S4Fig needs better definition

**Discussion:**

It would be very proper if the authors report the socio-economic characteristics of participants that are parents. This variable is important in the moment to make a decision when is shopping shoes. One limitations that is important to discuss is that the variability of foot feature is wide and it is influenced by the phenotype and social and economic characteristics the parents and children. For example, in Latin America is common when a family has a lot of children and has no enough money that younger children use the shoes of the older siblings, which can influence in the feature of foot of the younger sons.

It is important that authors discus about the main findings reported on S2 and S3 tables

**Conclusion:**

Without comments.
